# Global research on nanomaterials for liver cancer from 2004 to 2023: a bibliometric and visual analysis

**DOI:** 10.1007/s12672-024-01735-1

**Published:** 2024-12-26

**Authors:** Yitao Fan, Han Xiao, Yan Wang, Shuhan Wang, Hui Sun

**Affiliations:** 1 Cuiying Biomedical Research Center, The Second Hospital & Clinical Medical School, Lanzhou University, Lanzhou, 730030 Gansu China; 2 Lanzhou University, Lanzhou, 730030 Gansu China

**Keywords:** CiteSpace, VOSviewers, Bibliometrics, Nanomaterials, Nanoparticles, Liver cancer, Drug delivery

## Abstract

**Background:**

Primary liver cancer, particularly hepatocellular carcinoma, is one of the most common gastrointestinal cancers. An increasing number of studies indicate that nanomaterials play a significant role in the diagnosis and treatment of liver cancer. However, despite the extensive and diverse research on nanomaterials and liver cancer, bibliometric studies in this field have not yet been reported. This study aims to comprehensively evaluate the application prospects and development trends of nanomaterials in primary liver cancer over the past 20 years. By elucidating the current state of research on liver cancer, we intend to provide valuable reference information for researchers in this field.

**Methods:**

We conducted a comprehensive search of the Web of Science Core Collection for publications related to liver cancer and nanomaterials from January 1, 2004, to December 31, 2023. Relevant literature was selected based on specific inclusion and exclusion criteria. These selected publications were subsequently analyzed using CiteSpace, VOSviewer, and the R package "bibliometrix" to identify trends, influential countries, institutions, authors, journals, and research hotspots in this field.

**Results:**

This study included a total of 1641 publications, with an annual growth rate of 25.45%. China and the United States are leading in this field, accounting for 67.46% and 11.27% of the total publications, respectively. The Chinese Academy of Sciences and Shao D are the most cited institution and author, respectively. The International Journal of Nanomedicine is the most influential journal in this field, while Biomaterials is the most highly cited and co-cited journal. Research hotspots mainly focus on improving drug delivery efficiency, inducing cancer cell apoptosis, photodynamic therapy, photothermal therapy, and combination treatments. Emerging research directions include the tumor microenvironment, polyethylene glycol, and immunogenic cell death.

**Conclusion:**

The results of this study indicate that the application of nanomaterials in the field of liver cancer is gradually becoming a significant research area, with a focus on improving drug delivery efficiency, enhancing therapeutic efficacy, and reducing side effects.

## Introduction

Primary liver cancer is a type of malignant gastrointestinal cancer occurring in the liver, imposing a significant burden on the global economy and healthcare system. Hepatocellular carcinoma (HCC) accounts for 90% of primary liver cancer cases [1]. According to the "2022 Global Cancer Statistics Report" published by the International Agency for Research on Cancer under the World Health Organization [2], the incidence and mortality rates of liver cancer rank sixth and third worldwide, respectively, accounting for over 8% of total cancer-related deaths. Chronic infection with viral hepatitis, particularly hepatitis B, is the major risk factor for liver cancer, along with other factors such as smoking, alcohol consumption, aflatoxin, and obesity, which vary by region [3].

To date, treatment options for liver cancer include surgical resection, locoregional therapies, systemic chemotherapy, molecular targeted therapy, and immunotherapy. For advanced and metastatic liver cancer, immunotherapy is an indispensable treatment method. In the past five years, the primary focus of immunotherapy for HCC has been on immune checkpoint inhibitor (ICI). By blocking immune checkpoint pathways such as PD-1/PD-L1 and CTLA-4, ICI relieve the suppression of T cells, thereby restoring their antitumor functions [4]. In addition, ICI reshape the tumor microenvironment (TME) in HCC, enhancing the cytotoxic capacity of T cells. When used in combination with other therapies, ICI have significantly improved the outcomes for advanced liver cancer patients compared to traditional treatments such as sorafenib. Key advancements in immunotherapy include the use of CTLA-4-targeting agents such as ipilimumab and tremelimumab, PD-1 inhibitors such as pembrolizumab and nivolumab, and the combination of the PD-L1 inhibitor atezolizumab with the VEGF-targeting agent bevacizumab [4–7]. Notably, the combination of atezolizumab and bevacizumab has shown superior efficacy in improving overall survival and progression-free survival compared to traditional sorafenib treatment, becoming the new standard for first-line treatment of unresectable hepatocellular carcinoma (uHCC) [8]. Additionally, based on the positive results from the phase III HIMALAYA clinical trial, the US Food and Drug Administration approved the combination of tremelimumab and durvalumab for adult patients with uHCC. This combination therapy reduced the risk of death by 22% compared to sorafenib monotherapy [9]. Besides ICI, cytokine-induced killer cell immunotherapy has shown potential in treating liver cancer, along with lymphokine-activated killer cell therapy and chimeric antigen receptor T-cell (CAR-T) therapy, which can supplement standard treatment regimens for liver cancer. Given that most patients with liver cancer are diagnosed at an advanced stage, treatment options become significantly more challenging, and traditional therapeutic methods often prove less effective. Current treatment modalities, such as ICI, unlike conventional anticancer agents, typically do not exhibit immediate tumor-suppressing effects during the early stages of tumor progression [10]. This delayed response introduces unique challenges in clinical outcomes and may be accompanied by serious immune-related adverse events, including colitis, dermatitis, and hypophysitis [10–12]. Consequently, there is an urgent need to investigate and develop novel therapeutic strategies that can address these limitations.

Nanomaterials have been widely studied for their unique properties in precise cancer diagnosis and efficient treatment. These materials typically have diameters ranging from 1 to 100 nm. As detection and imaging agents, nanomaterials enable early diagnosis and precise localization of tumors or lesions, overcoming the limitations of traditional detection and imaging methods [13]. Additionally, nanomaterials can serve as drug carriers to achieve targeted and precise drug delivery, reducing side effects and drug resistance, and are used in photothermal therapy (PTT), photodynamic therapy (PDT), sonodynamic therapy (SDT), magnetic hyperthermia, and combination therapies. For example, titanium dioxide nanoparticles (TiO2 NPs) are suitable for tumor PDT and PTT therapy due to their remarkable photocatalytic activity [14]. RGD-conjugated gold NPs exhibit strong affinity for integrin receptors, which are highly expressed in cancer cells through the binding of RGD peptides. This specific interaction significantly enhances the targeting ability of these NPs at tumor sites, showing promising results in enhancing radiotherapy, drug delivery, and imaging [15]. ZIF-8 NPs, a type of metal–organic framework, are characterized by their high surface area, tunable pore sizes, and excellent stability. In specific applications, such as pH-responsive drug release within the tumor microenvironment, ZIF-8 NPs offer enhanced precision and control. Furthermore, by conjugating folic acid to target cancer cells that overexpress folate receptors, these NPs become highly suitable for targeted therapies requiring precise drug release regulation. This dual functionality, encompassing both environmental responsiveness and receptor targeting, makes ZIF-8 a promising platform for delivering chemotherapeutic agents like doxorubicin directly to tumor sites, improving treatment efficacy while minimizing off-target effects [16, 17]. In contrast, aptamer-functionalized carbon-based nanomaterials, with their high drug-loading capacity and flexible functionalization, are well-suited for various drug delivery applications [18]. Despite their potential, materials such as functionalized carbon-based nanomaterials tend to exhibit higher toxicity, especially when insufficiently functionalized. Therefore, the issue of toxicity caused by in vivo accumulation must be addressed to ensure the safety and efficacy of these materials in clinical applications.

**Fig. 1:**
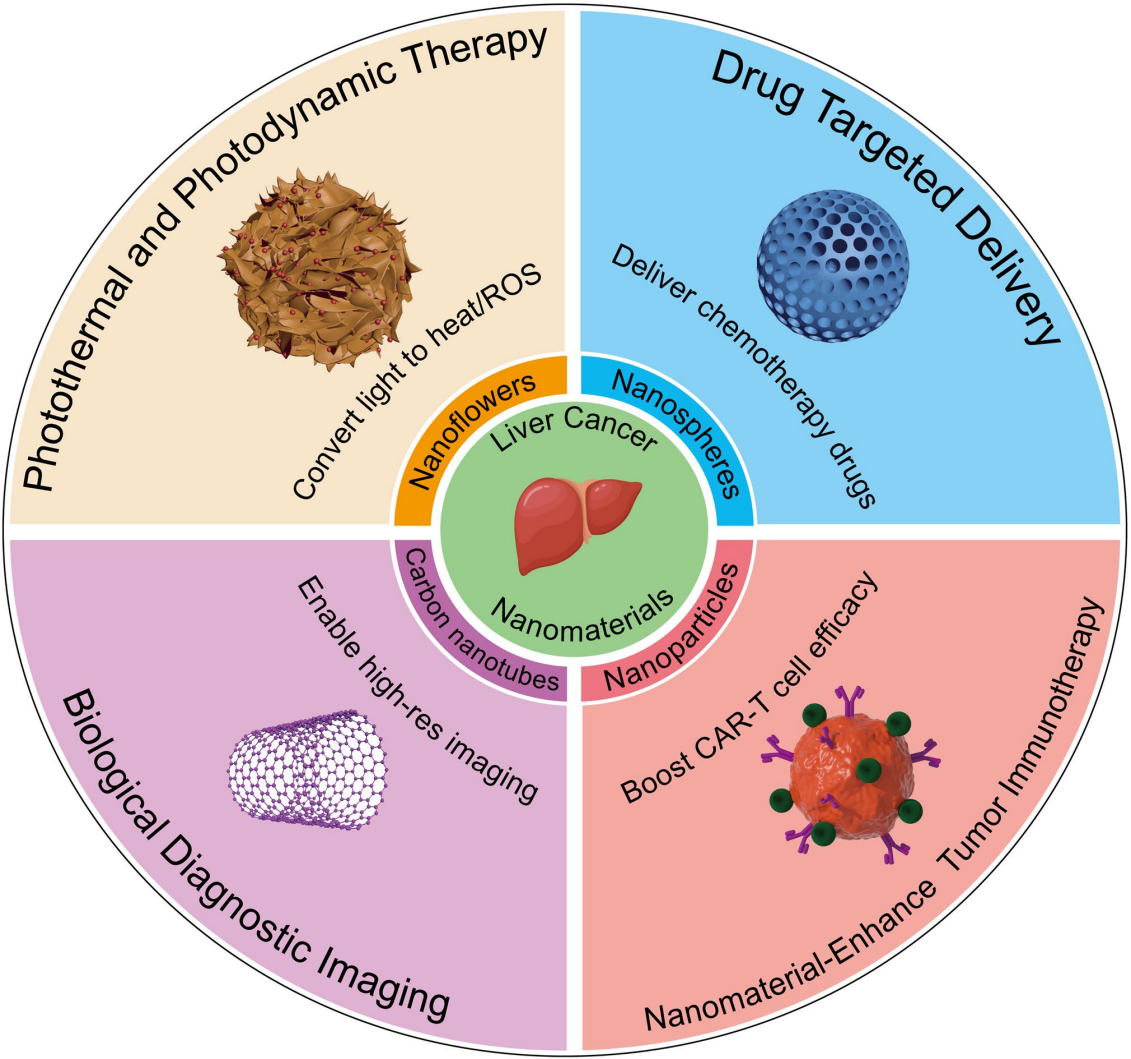
Multifunctional applications of nanomaterials in liver cancer treatment and diagnosis. The image illustrates four primary applications of nanomaterials in liver cancer treatment and diagnosis. These include: photothermal and photodynamic therapy, which converts light to heat or reactive oxygen species; drug-targeted delivery for chemotherapy drugs; biological diagnostic imaging enabled by carbon nanotubes for high-resolution imaging; and nanomaterial-enhanced tumor immunotherapy, boosting CAR-T cell efficacy. Various forms of nanomaterials, such as nanoflowers, nanospheres, and nanoparticles, play crucial roles in these applications

The rapid development of nanotechnology has introduced nanomaterials with unique properties into the biomedical field, particularly in the management of HCC (Fig. 1). Traditional imaging methods, such as magnetic resonance imaging (MRI) and computed tomography (CT), exhibit limitations in diagnosing early-stage HCC. Therefore, novel nanomaterials, such as superparamagnetic iron oxide nanoparticles (SPIONs), have been developed as more efficient MRI contrast agents compared to traditional ones, offering improved imaging of the hepatobiliary system [19, 20]. Additionally, the integration of nanotechnology, such as nanobubbles for ultrasound imaging, enables localized drug release under ultrasound guidance, which plays a significant role in the early diagnosis of HCC [21, 22]. In liver cancer treatment, nanotechnology-based drug delivery systems (NDDS) have demonstrated substantial improvements over conventional therapies like sorafenib, which is hindered by challenges such as poor solubility, rapid metabolism, and non-specific distribution. NDDS enhances the stability, solubility, and bioavailability of the drug, overcoming these limitations [23]. Polymeric NPs, including poly (lactic acid) (PLA) and poly (lactic-co-glycolic acid) (PLGA) NPs, as well as lipid-based NPs like liposomes and solid lipid NPs, are frequently used to encapsulate sorafenib. This encapsulation allows for controlled and sustained drug release, maintaining therapeutic drug concentrations over extended periods while reducing toxicity to normal tissues [24–26]. Furthermore, NDDS utilizes the enhanced permeability and retention (EPR) effect, facilitating passive accumulation of sorafenib in tumor tissues and increasing its concentration at the tumor site [27, 28]. This targeted delivery approach not only reduces systemic side effects but also optimizes drug concentration in the tumor microenvironment, effectively lowering drug resistance and ultimately improving patient outcomes. Given the potential of nanotechnology in the diagnosis and treatment of liver cancer, an increasing number of studies are exploring its applications, highlighting the need for a comprehensive analysis of this research area.

Modern bibliometrics, first proposed by Derek J. de Solla Price in the late 1950s and early 1960s, has gradually evolved into a branch of information science through continuous innovation and expanded applications. Its core involves the application of mathematical and statistical methods to quantitatively analyze the citation and publication of scientific literature, identifying influential countries, journals, institutions, authors, and frequently cited publications, references, and keywords [29, 30]. Additionally, bibliometrics generates visual representations, such as collaboration networks between countries, institutions, and authors, allowing researchers to understand the developmental history and frontiers of a specific field [31]. By quantifying trends, identifying key authors, and recognizing influential papers, bibliometric analysis provides not only a comprehensive and in-depth overview of the research landscape but also reveals the core issues and directions in the field. This method helps guide future research and optimizes the allocation of academic resources as well as the construction of collaboration networks.

To our knowledge, no bibliometric analysis has yet focused on the application of nanomaterials in the field of liver cancer, highlighting a critical gap in interdisciplinary research between materials science, nanotechnology, and oncology. By bridging these disciplines, this study provides a comprehensive overview of the role of nanomaterials in the diagnosis and treatment of liver cancer. We conducted a bibliometric analysis of the literature from the past twenty years, offering the first systematic evaluation of research trends in this area, and identifying the most influential papers and landmark studies. This analysis not only reveals under-researched areas within the field, encouraging further studies, but also deepens our understanding of the intersections between nanotechnology, liver cancer, and clinical applications. In addition, it objectively evaluates the scientific contributions of various scholars and institutions. By identifying key research clusters and collaborative networks, this study provides valuable guidance for future interdisciplinary research, driving innovation in the diagnosis and treatment of liver cancer.

## Materials and methods

### Information sources and retrieval

As one of the most commonly used high-quality academic resources by researchers and academic institutions, the Web of Science Core Collection (WoSCC) is considered the best database for bibliometric analysis [32, 33]. Therefore, we chose to use WoSCC for our search. This study retrieved all articles related to liver cancer and nanomaterials, covering the time span from January 1, 2004, to December 31, 2023. Since the WoSCC topic search is based on the overall retrieval of article titles, abstracts, authors, and keywords, we used topic searches to ensure accuracy [34].

The retrieval approach is as follows: Query #1 Topic = ("Liver Cancer" OR "Liver Cancers" OR "Liver Carcinoma" OR "Liver Carcinomas" OR "Liver Neoplasm" OR "Liver Neoplasms" OR "Liver Tumor" OR "Liver Tumors" OR "Liver Malignancy" OR "Hepatic Cancer" OR "Hepatocellular Carcinoma" OR "Hepatoma" OR "Liver Cell Carcinoma" OR "Primary Liver Cancer" OR "Cancer of the Liver" OR "Cancer of Liver"). Query #2 Topic = (nano*), where * is a wildcard that retrieves all different spelling forms of nano. Search #1 AND #2.

### Selection criteria

The inclusion criteria are as follows: (1) The article focuses on liver cancer and nanomaterials, and the full text is available. (2) The publication type is article or review article. (3) The time span is from January 1, 2004, to December 31, 2023. (4) The article is written in English. (5) The literature is sourced from the WoSCC. The exclusion criteria are as follows: (1) The article's topic is unrelated to liver cancer and nanomaterials or cannot be evaluated. (2) The publication type is meeting abstract, dissertation, proceeding paper, editorial material, News item, etc. (3) The time range does not meet the requirements. (4) The article is not written in English. (5) The literature is not sourced from the WoSCC.

Two authors independently assessed the full text against the inclusion and exclusion criteria. Any disagreements between the two authors (Fan Yt and Xiao H) were resolved through discussion with a third author (Sun H). For the included publications, data were exported in plain text file, including full records and cited references. We extracted the following variables from the publications: annual publication count, country/region, institution, author, journal, title, keywords, and co-cited references. Additionally, the 2023 edition of the Journal Citation Reports (JCR), impact factor (IF), and H-index were included in the analysis as key indicators to measure the scientific value of the research.

### Data analysis tools and techniques

All data in this study were processed and visualized using CiteSpace software (Version advanced 6.2.R3 Pro), VOSviewer software (Version 1.6.18), the Bibliometrix R package (Version 4.3.3), and GraphPad Prism (Version 9.4).

CiteSpace, developed by Professor Chen CM on the Java platform, is a tool for visually exploring trends and patterns in scientific literature. It identifies "burst citations" for trend analysis, constructs co-citation networks, provides temporal and spatial views, and uses clustering techniques to identify research themes and trends [35]. In this study, CiteSpace was used to create a co-authorship network map of countries and institutions, a dual-map overlay of journals, a cluster analysis of co-cited references, and to display citation bursts. The parameters used were time slicing from January 2004 to December 2023, with one-year intervals. The selection used a modified g-index in each slice: g2 ≤ k∑i ≤ gci,k ∈ Z + , with a scale factor *k* = 25 to include more or fewer nodes. The top 50 levels of the most cited or occurred items from each slice were selected. Node size indicates the frequency of citations or occurrences. The connections between nodes represent collaboration and co-citation relationships. Different colored nodes represent different years. In CiteSpace clustering analysis, the key parameters include Modularity (Q) and Weighted Mean Silhouette (S) values. Q values greater than 0.3 indicate a significant clustering structure. S values above 0.5 are considered acceptable for clustering, and values over 0.7 suggest the clustering results are highly reliable [36].

VOSviewer is a tool specifically designed for constructing and viewing bibliometric maps. It offers literature citation networks and co-citation analysis features, supported by embedded clustering algorithms [37]. It is widely used in the analysis and management of scientific literature. In this study, VOSviewer was primarily used for visualizing the density of citations for countries and institutions, and for conducting visual analysis of authors and journals. This included constructing co-authorship network map, timeline network maps, and co-citation network maps.

Bibliometrix is an open-source tool developed by Aria M and Cuccurullo C for quantitative research in scientometrics and bibliometrics [38]. It includes all major bibliometric analysis methods. Through importing bibliographic data from various databases, Bibliometrix performs bibliometric analyses and constructs data matrices for co-citation, coupling, collaboration, and co-word analysis. This tool facilitates systematic, transparent, and reproducible reviews, helping to track and utilize the rapidly increasing volume of academic publications. In this study, Bibliometrix was primarily used for the visualization of keywords. This included creating word cloud of keywords and trend chart showing the evolution of keywords over the years.

## Results

### Annual trends analysis of publications on nanomaterials in liver cancer

**Fig. 2:**
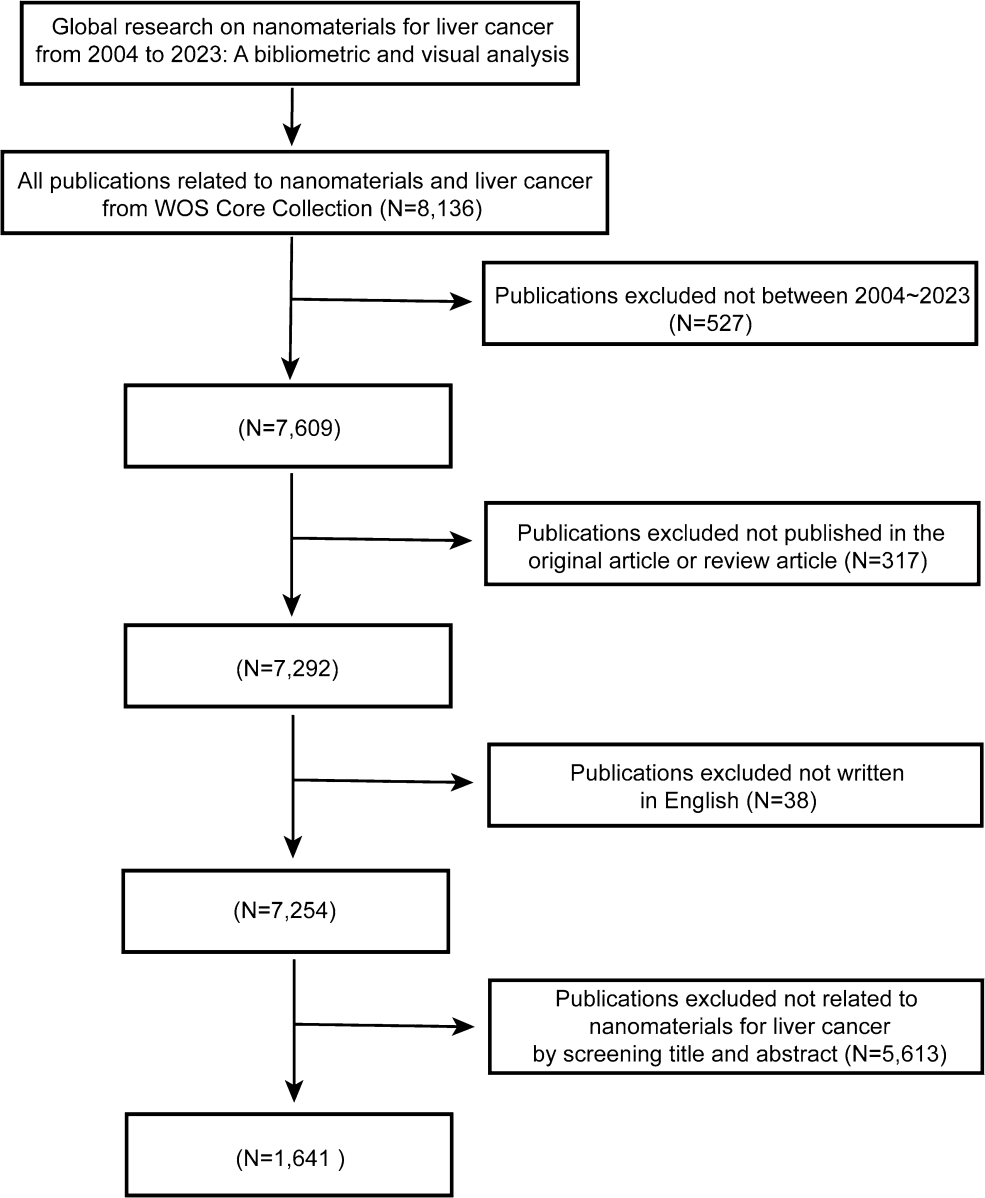
Flowchart displaying the progress of publications selection

From January 1, 2004, to December 31, 2023, a total of 8136 publications were retrieved from the WoSCC, spanning a period of 20 years. After further screening based on specific inclusion and exclusion criteria, 1641 publications were included (Fig. 2).

**Fig. 3:**
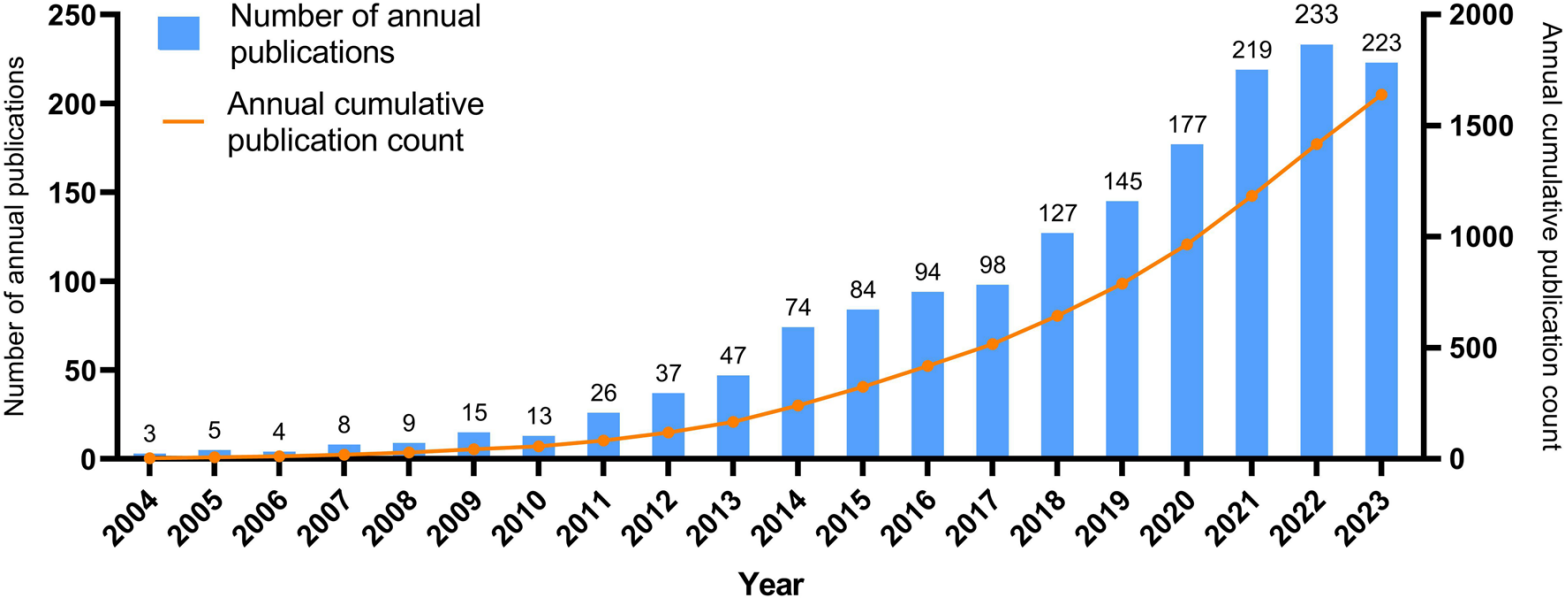
The annual and cumulative trends in publications on nanomaterials for liver cancer from 2004 to 2023

These publications originate from 64 different countries or regions, 1714 institutions, and 9148 authors. A total of 215 institutions published five or more publications related to liver cancer and nanomaterials. These publications were published in 473 different journals, including 1552 articles (percentage = 94.58%) and 89 review articles (percentage = 5.42%). The annual number of publications on liver cancer and nanomaterials from 2004 to 2023 is shown in Fig. 3. We divided this period into four phases: a slow growth phase from 2004 to 2010, a steady rise phase from 2011 to 2017, a rapid growth phase from 2018 to 2021, and a stagnation phase from 2022 to 2023, with an annual growth rate of 25.45%. Notably, the number of publications in 2022 was the highest in the past two decades. Additionally, Table 1 shows the total number of citations and the citation per publication for annual publications from 2004 to 2023. The highest citation per publication was in 2009 (138.80), while the highest total number of citations was in 2018 (4699). Overall, the growth in publication count was slow and steady before 2017, followed by an explosive increase starting in 2018. However, from 2022 to 2023, the growth in publication count slowed down, with a slight decrease in 2023. Despite this, the overall interest and focus on nanomaterials in the field of liver cancer have been increasing year by year.

**Table 1:** Annual publication and citation statistics on nanomaterials for liver cancer research from 2004 to 2023

Year	Publication count	Percentage (N/1641)	Citation	Citation per publication
2004	3	0.183	106	35.33
2005	5	0.305	264	52.80
2006	4	0.244	311	77.75
2007	8	0.488	367	45.88
2008	9	0.548	319	35.44
2009	15	0.914	2082	138.80
2010	13	0.792	1176	90.46
2011	26	1.584	2214	85.15
2012	37	2.255	2494	71.26
2013	47	2.864	2230	47.45
2014	74	4.509	3097	41.85
2015	84	5.119	3286	39.59
2016	94	5.728	3550	37.77
2017	98	5.972	3252	33.18
2018	127	7.739	4699	37.00
2019	145	8.836	4588	31.86
2020	177	10.786	4060	22.94
2021	219	13.346	3402	15.53
2022	233	14.199	1978	8.49
2023	223	13.589	652	2.92

### Analysis of contributions by countries and regions

**Fig. 4:**
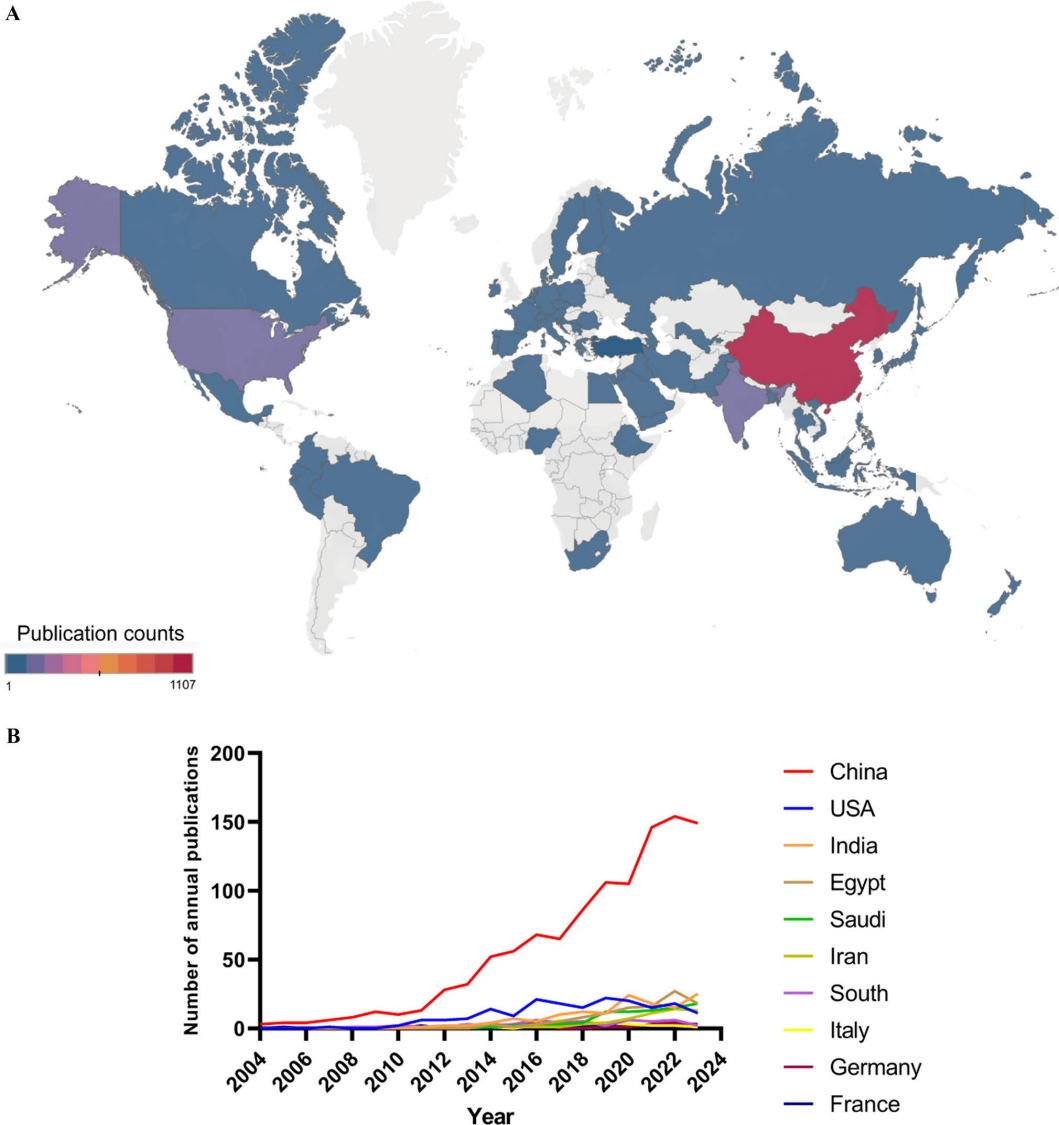

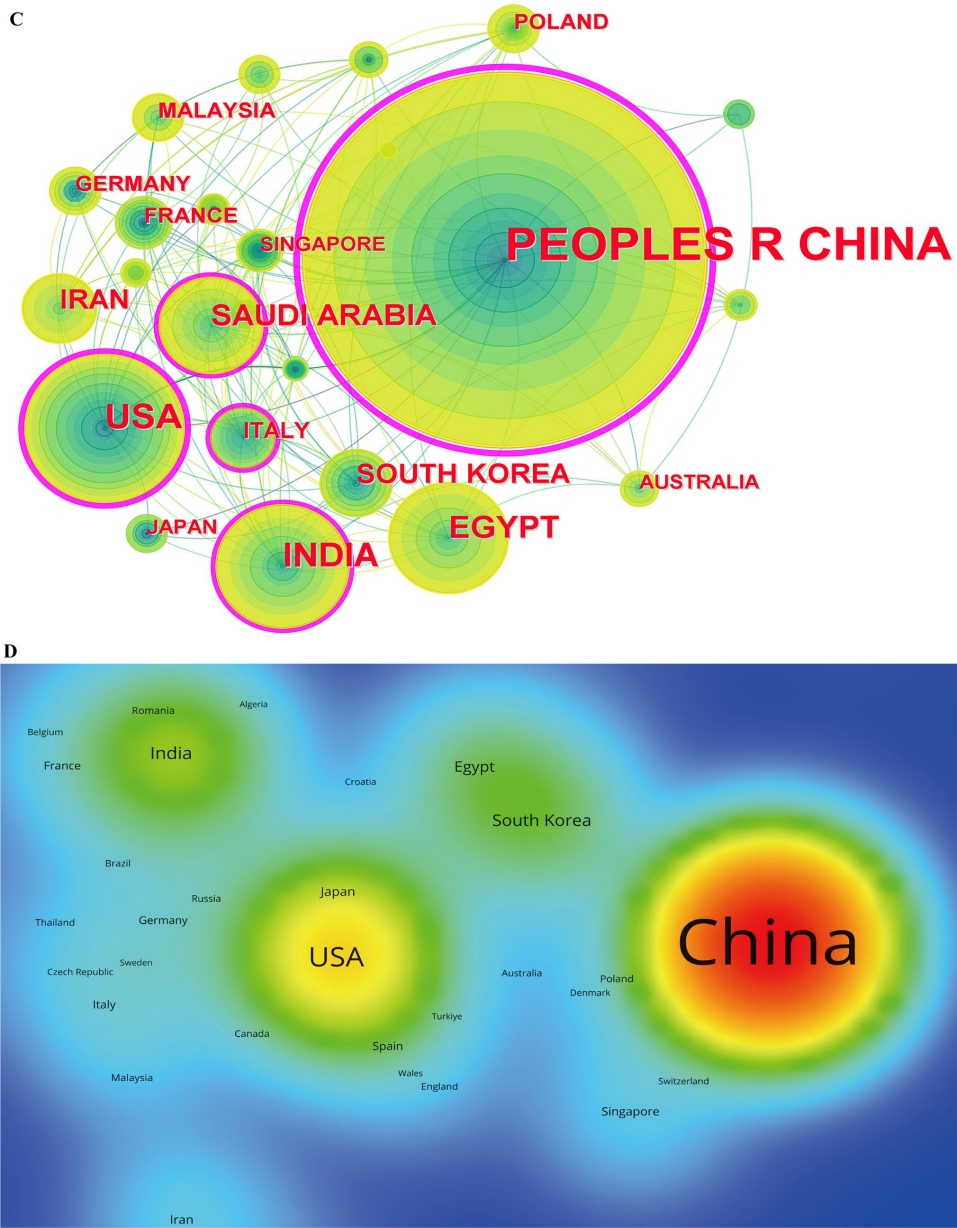
The visualization of countries/regions involved in liver cancer and nanomaterials research. **A** Distribution of countries/regions based on the number of publications. **B** Annual publication trends of top 10 countries from 2004 to 2023. **C** Co-authorship network map of countries/regions. Countries/regions are represented as nodes, and cooperative relationships are shown as connecting lines. The node area increases with the number of publications. The internal color of the nodes represents different publication years. The color of the connecting lines reflects the years of cooperation between countries, changing from dark green to bright yellow from 2004 to 2023. Some nodes have a purple outer ring, indicating higher centrality within the cooperative network. **D** Density of citations by countries/regions. The size and brightness of the nodes represent citation frequency. Red indicates the highest citation frequency, while blue indicates the lowest

As shown in Fig. 4A, a total of 64 different countries or regions have published literature related to liver cancer and nanomaterials. Table 2 and Fig. 4B exhibit the top 10 most prolific countries in this field.

**Table 2:** The top 10 most prolific countries on nanomaterials for liver cancer research

Rank	Country/Region	Publication count	Percentage (N/1,641)	Citation	Citation per publication	Centrality	H-index
1	China	1107	67.46	29,197	26.37	0.58	75
2	USA	185	11.27	7199	38.91	0.15	51
3	India	136	8.29	2792	20.53	0.22	30
4	Egypt	107	6.52	1922	17.96	0.12	26
5	Saudi Arabia	83	5.06	1060	12.77	0.20	19
6	Iran	58	3.53	1014	17.48	0.24	18
7	South Korea	50	3.05	2391	47.82	0.02	23
8	Italy	26	1.58	757	29.12	0.15	17
9	Germany	23	1.40	608	26.43	0.03	13
10	France	22	1.34	712	32.36	0.02	13

China has published the most papers in this field (n = 1107), accounting for 67.46% of the total. It is followed by the United States (n = 185, 11.27%), India (n = 136, 8.29%), Egypt (n = 107, 6.52%), and Saudi Arabia (n = 83, 5.06%). China's annual publication count far exceeds that of other countries. Although China has the highest total publication count (Fig. 4C) and the most citations (29,197) (Fig. 4D), its citation per publication (26.37) is lower than that of the United States (38.91), ranking sixth (Table 2). Notably, China's centrality (0.58) and H-index (75) are higher than those of other countries (Table 2). Centrality indicates the bridging role and importance of a node in the network, with high centrality nodes typically representing key countries or research institutions in academic communication and knowledge dissemination [39]. The H-index effectively reflects the academic output and impact of a country, scholar, or research institution. These indicators collectively show that China has made stable scientific contributions and academic output in the field of liver cancer and nanomaterials over a long period. Additionally, among these ten countries, South Korea, although having a lower publication count, has the highest citation per publication (47.82), indicating its research output's academic potential and significance in this field.

Furthermore, the co-authorship network map of countries/regions (Fig. 4C) also shows the collaborative relationships of publications between each country. The color of the connecting lines ranges from light to dark, representing the time from recent to past. For instance, in recent years, China has collaborated with Poland, Turkey, and Iraq in the field of liver cancer and nanomaterials, while the United States has collaborations with Egypt, Turkey, and Portugal.

### Analysis of contributions by institutions

In the field of liver cancer and nanomaterials, a total of 1714 institutions have contributed. Table 3 shows the top 10 institutions by publication count, accounting for 34.06% of the total publications. Among them, nine institutions are from China, indicating a high level of attention to this field in China. The Egyptian Knowledge Bank published the most papers (n = 106, 6.46%), followed by the Chinese Academy of Sciences (n = 103, 6.28%), Zhejiang University (n = 63, 3.84%), Sun Yat-sen University (n = 60, 3.66%), and Huazhong University of Science and Technology (n = 51, 3.11%).

**Table 3:** The top 10 most prolific institutions on nanomaterials for liver cancer research

Rank	Institution	Country	Publication count	Percentage (N/1,641)	Citation	Citation per publication	Centrality	H-index
1	Egyptian Knowledge Bank EKB	Egypt	106	6.46	1921	18.12	0.28	26
2	Chinese Academy of Sciences	China	103	6.28	4388	42.60	0.33	38
3	Zhejiang University	China	63	3.84	1772	28.13	0.09	21
4	Sun Yat-sen University	China	60	3.66	1466	24.43	0.09	23
5	Huazhong University of Science Technology	China	51	3.11	1499	29.39	0.22	22
6	Fudan University	China	41	2.50	1384	33.76	0.08	18
7	Jilin University	China	41	2.50	1500	36.59	0.01	24
8	Southern Medical University	China	38	2.32	789	20.76	0.03	16
9	Shanghai Jiao Tong University	China	37	2.26	1182	31.95	0.09	18
10	University of Chinese Academy of Sciences UCAS	China	35	2.13	1253	35.80	0.02	18

**Fig. 5:**
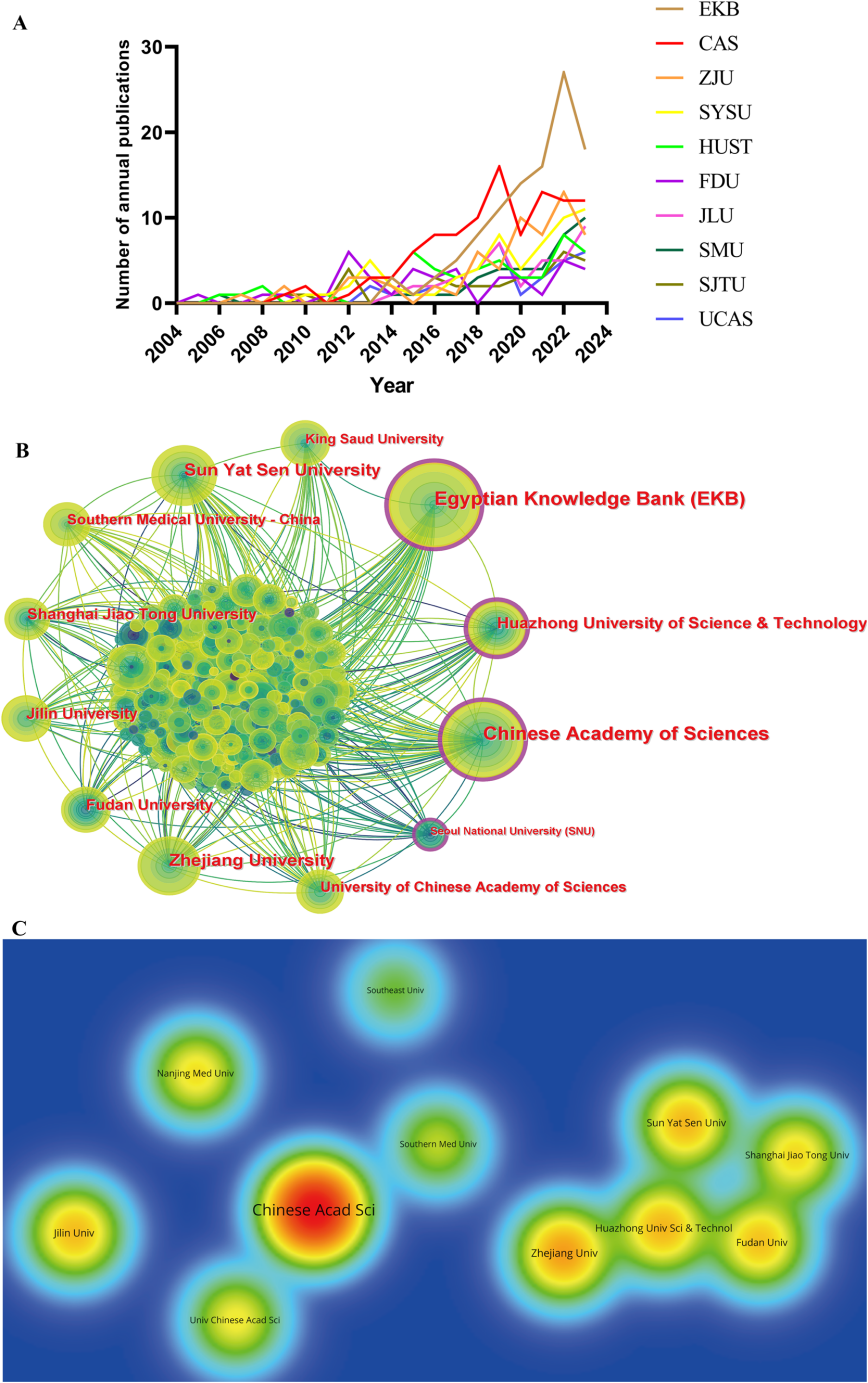
The visualization of institutions involved in liver cancer and nanomaterials research. **A** Annual publication trends of top 10 institutions from 2004 to 2023. **B** Co-authorship network map of institutions. Institutions are represented as nodes, and cooperative relationships are shown as connecting lines. The node area increases with the number of publications. The internal color of the nodes represents different publication years. The color of the connecting lines reflects the years of cooperation between institutions, changing from dark green to bright yellow from 2004 to 2023. Some nodes have a purple outer ring, indicating higher centrality within the cooperative network. **C** Density of citations by institutions. The size and brightness of the nodes represent citation frequency. Red indicates the highest citation frequency, while green indicates the lowest

In terms of annual publication count (Fig. 5A), the Egyptian Knowledge Bank's publications have steadily increased since 2014, with a sharp rise in 2021 and 2022, followed by a decline in 2023. The Chinese Academy of Sciences, ranked second, has shown growth since 2009, peaking in 2019, and fluctuating in subsequent years. Overall, most institutions saw a peak in publication numbers in 2022, followed by a decline in 2023 (Fig. 5A). Although the Egyptian Knowledge Bank has the highest number of publications (Fig. 5B), its total citations (1921) and citation per publication (18.12) are relatively low (Table 3). The Chinese Academy of Sciences, although ranked second in publication count, has the highest total citations (4388) (Fig. 5C), citation per publication (42.60), centrality (0.33), and H-index (38) (Table 3). This indicates that the research outputs of the Chinese Academy of Sciences in the field of liver cancer and nanomaterials are widely recognized and cited, reflecting significant scientific value and academic impact. Notably, although Seoul National University has a lower publication count, it has a high centrality (0.11), indicating its crucial role in academic exchange and strategic collaboration. In recent years, the Egyptian Knowledge Bank has collaborated extensively with institutions such as Fayoum University, Jazan University, and King Abdulaziz University Hospital, while the Chinese Academy of Sciences has collaborated with Wenzhou Medical University, Anhui Medical University, and Hefei University of Technology.

### Analysis of contributions by authors and cited authors

**Fig. 6:**
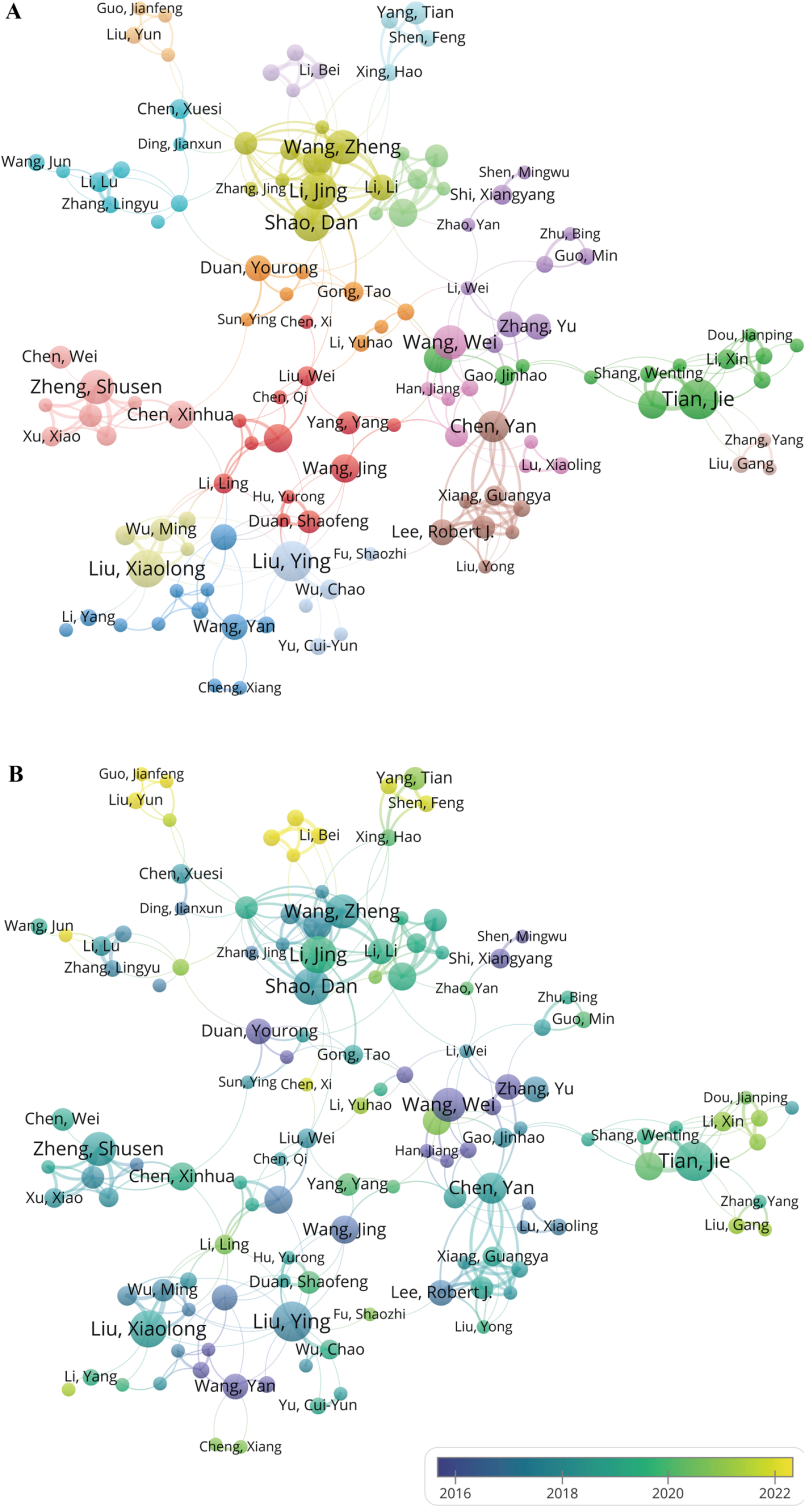

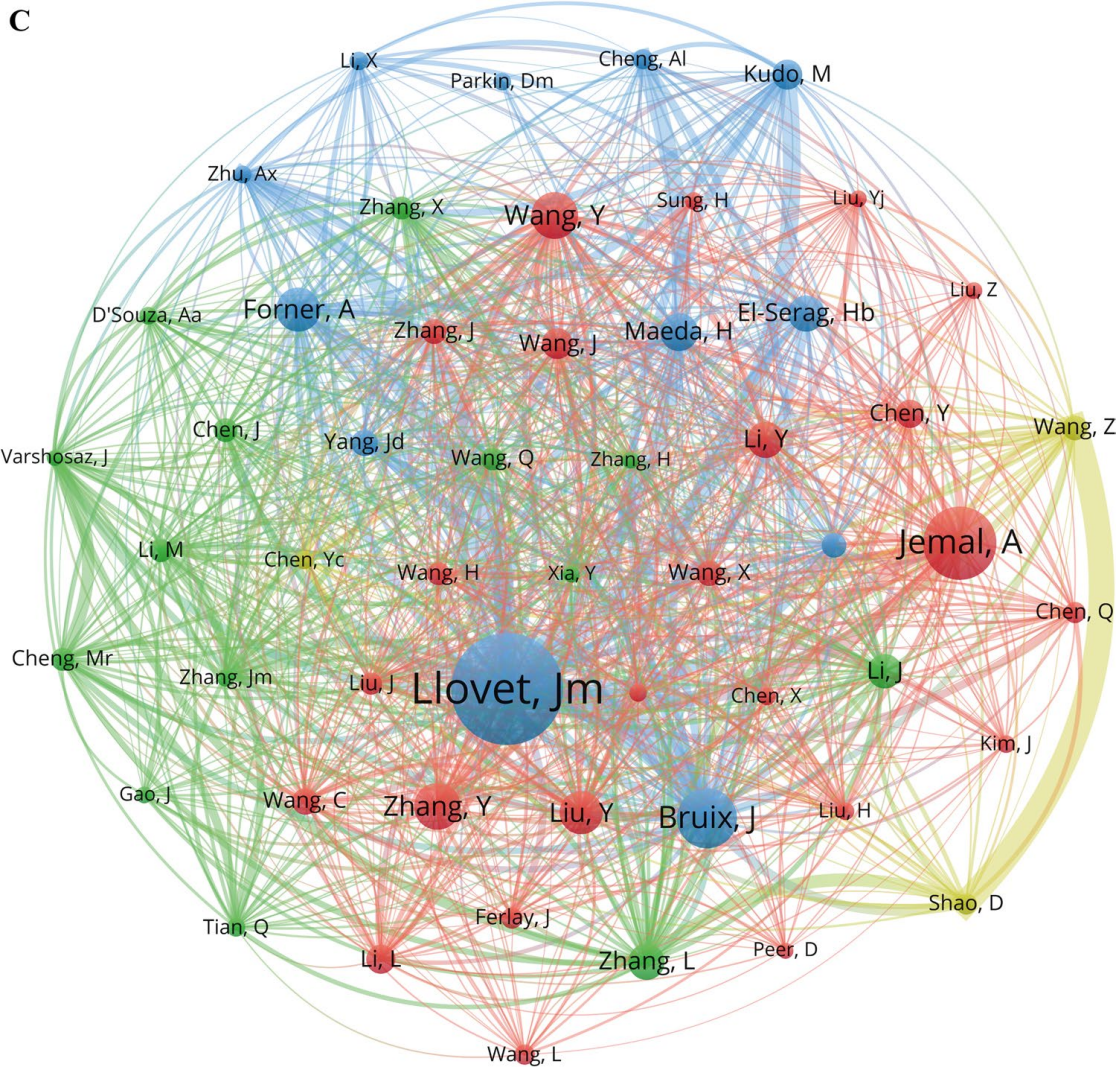
The visualization of authors and co-cited authors involved in liver cancer and nanomaterials research. **A** Co-authorship network map of authors. The connecting lines between nodes represent the strength of collaboration; the thicker the line, the stronger the collaboration. The area of each node is proportional to the number of publications, with larger nodes indicating a higher publication count. Different clusters are represented by different colors. These colors indicate groups of authors who are more closely related to each other in terms of their collaborative relationships. Each cluster represents a set of authors who frequently co-author papers together, suggesting a higher degree of collaboration within the cluster compared to authors outside the cluster. **B** Timeline network map of authors. **C** Co-citation network map of authors. The size of the nodes indicates the frequency of citations for each author; larger nodes represent higher citations. Different colored nodes represent distinct clusters, indicating strong associations between these authors. The lines between nodes signify co-citation relationships; more and thicker lines denote a higher frequency of simultaneous citations for the two authors

In bibliometrics, author analysis plays a crucial role. It not only helps identify the most significant contributors in a specific field but also reveals the overall structure and development trends of academic research. Over the past twenty years, a total of 9148 authors have contributed to the field of liver cancer and nanomaterials. Table 4 shows the top 10 authors with the highest number of publications. Liu Y and Tian J have published the most articles in this field (n = 14), followed by Li J (n = 13), Liu XL (n = 13), and Shao D (n = 13). All ten authors are from China. Among them, Shao D has the highest citation per publication (69.85) and H-index (13). We constructed a co-authorship network by analyzing authors who have published five or more papers (Fig. 6A). Shao D has collaborated with other authors the most frequently (Total Link Strength, TLS = 58), particularly with Wang Z (Link Strength, LS = 11). Further analysis using a timeline network map (Fig. 6B) indicates that the collaboration between Shao D and Wang Z was most active from 2017 to 2019, and both were affiliated with the Chinese Academy of Sciences. In recent years, emerging authors such as Li B, Liu Y, and Guo JF have also been increasing their publication count (Fig. 6B). Overall, the research in the field of liver cancer and nanomaterials has begun to form a collaborative network among scholars, but the current cooperative relationships remain relatively loose.

**Table 4:** The top 10 most prolific authors and co-cited authors on nanomaterials for liver cancer research

Rank	Author	Country	Publication count	Percentage(N/1,641)	Citation	Citation per publication	H-index	Co-cited author	Country	Co-citation	Centrality
1	Liu, Y	China	14	0.85	498	35.57	12	Llovet, JM	Spain	348	0.07
2	Tian, J	China	14	0.85	462	33.00	10	Jemal, A	USA	227	0.02
3	Li, J	China	13	0.79	363	27.92	8	Bruix, J	Spain	186	0.06
4	Liu, XL	China	13	0.79	500	38.46	9	Wang, Y	China	145	0.03
5	Shao, D	China	13	0.79	908	69.85	13	Zhang, Y	China	145	0.05
6	Wang, W	China	12	0.73	627	52.25	10	Forner, A	Spain	139	0.02
7	Wang, Z	China	12	0.73	736	61.33	11	Liu, Y	China	134	0.01
8	Zheng, SH	China	12	0.73	335	27.92	9	Maeda, H	Japan	119	0.08
9	Chen, Y	China	11	0.67	390	35.45	9	Zhang, L	China	114	0.03
10	Zhang, J	China	11	0.67	169	15.36	8	El-Serag, HB	USA	114	0.07

Author co-citation refers to the situation where two or more authors are cited together in third-party literature. If author A and author B are frequently cited together in many documents, then there is a strong co-citation relationship between these two authors [40, 41]. In this study, a total of 37,357 authors were co-cited. Table 4 also shows the top 10 authors with the highest co-citation counts. Although these highly co-cited authors may not specialize in the field of nanomaterials for liver cancer diagnosis and treatment, their contributions have undoubtedly had a significant impact on the development of this field. Using a minimum of 50 citations per author as the threshold, we generated an author co-citation network to identify highly cited scholars (Fig. 6C). By analyzing author co-citation relationships, we can uncover the implicit academic connections and research networks among different authors. As seen in Table 4 and Fig. 6C, Lloret, JM is shown as a large blue node at the center, indicating that he is an author with very high co-citations in this field (348) and has the strongest co-citation network (TLS = 4229), with a close co-citation relationship with many authors, particularly Bruix, J (LS = 142). Following him are Jemal, A (227) and Bruix, J (186), who also appear as large nodes, indicating their significant influence in this field and their co-citation relationships with multiple authors. Notably, there is a substantial co-citation relationship between Shao D and Wang Z (LS = 106), indicating their research has high relevance and complementarity in academia, and their contributions in this field are significant. Additionally, authors with high centrality, such as Maeda, H and El-Serag, HB, have also made significant contributions to the development of this field (Table 4). Their research is widely cited and disseminated, playing a key role in the dissemination of knowledge and information flow in the academic community.

### Analysis of contributions by journals and co-cited journals

**Fig. 7:**
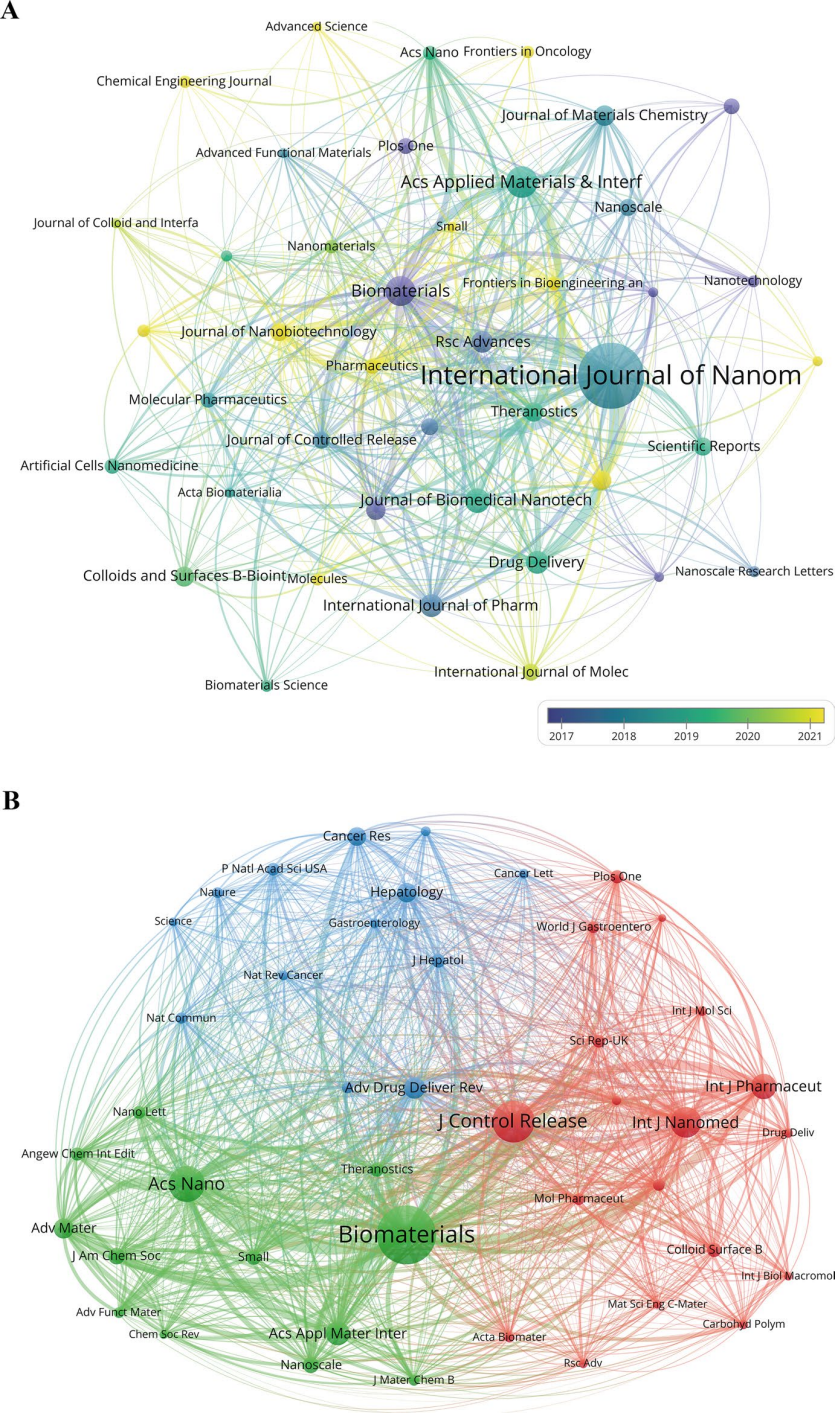

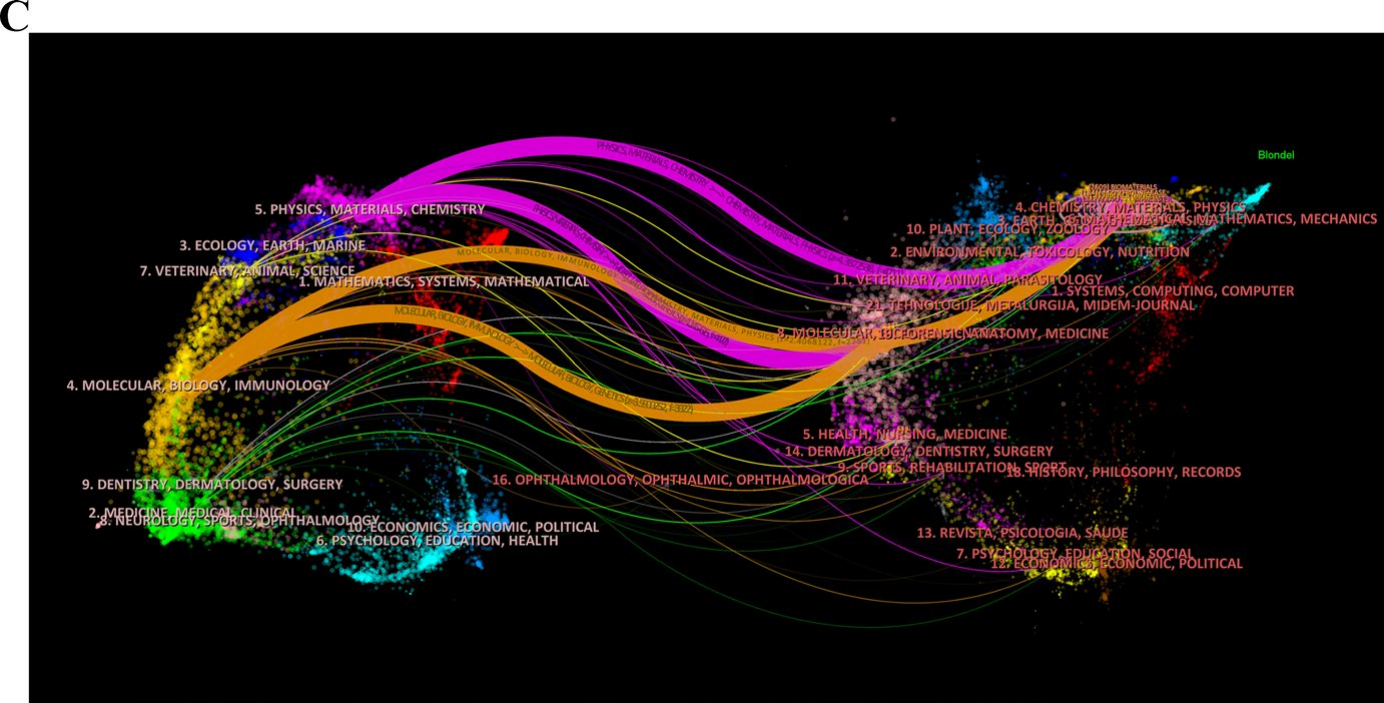
The visualization of journals and co-cited journals involved in liver cancer and nanomaterials research. **A** Timeline network map of journals. **B** Co-citation network map of journals. The size of the nodes indicates the frequency of citations for each co-journal; larger nodes represent higher citation counts. Different colored nodes represent distinct clusters, indicating strong associations between these journals. The lines between nodes signify co-citation relationships; more and thicker lines denote a higher frequency of simultaneous citations for the two journals. **C** Dual-map overlay of journals. The citing journals are positioned on the left, and the cited journals are on the right, with colored paths indicating citation relationships. The color and thickness of the paths represent the strength and frequency of the citations

The 1641 articles retrieved in this study were published in 473 different journals. Table 5 shows the top 12 journals by publication count in the field of liver cancer and nanomaterials, accounting for 22.85% of all publications. Among them, the International Journal of Nanomedicine published the most articles (n = 90, 5.48%), far exceeding other journals. It was followed by ACS Applied Materials & Interfaces (n = 38, 2.32%), Biomaterials (n = 35, 2.13%), Journal of Biomedical Nanotechnology (n = 28, 1.71%), Drug Delivery (n = 26, 1.58%), and International Journal of Pharmaceutics (n = 26, 1.58%). Among these top 12 most prolific journals, Biomaterials has the highest impact factor (2023 IF = 12.8), the highest number of citations (2926), and the highest citation per publication (83.60). The International Journal of Nanomedicine has the highest H-index (32), followed by Biomaterials (26) and ACS Applied Materials & Interfaces (24). It is important to note that the H-index here represents the H-index displayed under specific retrieval methods, limited to a specific range, and does not reflect the overall H-index of the journals. Additionally, 75% of the top 12 journals by publication count are classified as Q1 (based on JIF quartiles, i.e., the top 25% of journals by impact factor). These journals are considered the most influential and authoritative in their respective fields, indicating that the application of nanomaterials in liver cancer is widely recognized by high-quality journals, making it an active and important research area. Subsequently, we chose 42 journals, each with a minimum of 10 pertinent publications, to create a visualization in the form of a journal timeline network (Fig. 7A). The Fig. 7A shows that journals like the International Journal of Nanomedicine, ACS Applied Materials & Interfaces, and Biomaterials had the majority of their publications in the field of liver cancer and nanomaterials concentrated between 2017 and 2020. In recent years, emerging journals such as the Journal of Nanobiotechnology, Pharmaceutics, and Advanced Science have also begun to show their presence in the field of liver cancer and nanomaterials.

**Table 5:** The top 12 most prolific journals on nanomaterials for liver cancer research

Rank	Journal	Publication count	Percentage (N/1641)	Citation	Citation per publication	H-index	IF (2023)	JCR partition
1	International Journal of Nanomedicine	90	5.48	2757	30.63	32	6.6	Q1
2	ACS Applied Materials & Interfaces	38	2.32	1591	41.87	24	8.3	Q1
3	Biomaterials	35	2.13	2926	83.60	26	12.8	Q1
4	Journal of Biomedical Nanotechnology	28	1.71	487	17.39	14	2.9 (2022)	Q3 (2022)
5	Drug Delivery	26	1.58	724	27.85	17	6.5	Q1
6	International Journal of Pharmaceutics	26	1.58	966	37.15	16	5.3	Q1
7	RSC Advances	24	1.46	393	16.38	13	3.9	Q2
8	Journal of Materials Chemistry B	23	1.40	631	27.43	16	6.1	Q1
9	Colloids and Surfaces B:Biointerfaces	22	1.34	573	26.05	13	5.4	Q1
10	Journal of Drug Delivery Science and Technology	21	1.28	166	7.90	7	4.5	Q1
11	Nanomedicine-Nanotechnology Biology and Medicine	21	1.28	851	40.52	14	4.2	Q2
12	Theranostics	21	1.28	1124	53.52	19	12.4	Q1

Co-citation relationships between journals can reveal which journals are closely related within the same research theme or field. If two journals are frequently cited together in the same paper, they typically cover similar research content or areas. In this study, there are a total of 5950 co-cited journals. Table 6 shows the top 10 most co-cited journals in the field of nanomaterials for liver cancer. The most co-cited journal is Biomaterials (2610), followed by the Journal of Controlled Release (1843) and ACS Nano (1586). Advanced Materials has the highest impact factor (2023 IF = 27.4), followed by ACS Nano (2023 IF = 15.8) and Advanced Drug Delivery Reviews (2023 IF = 15.2). All ten of these co-cited journals are classified as Q1. Additionally, using a minimum threshold of 350 citations per source, we created a co-citation network map of publications based on co-cited journals (Fig. 7B). As shown in the figure, Biomaterials has the strongest co-citation network (TLS = 69,797), with particularly strong co-citation relationships with the Journal of Controlled Release (LS = 5462) and ACS Nano (LS = 4448). This indicates that articles from these journals are considered foundational in this research area and are of significant reference value for subsequent studies.

**Table 6:** The top 10 most co-cited journals on nanomaterials for liver cancer research

Rank	Co-cited journal	Co-citation	IF (2023)	JCR partition
1	Biomaterials	2610	12.8	Q1
2	Journal of Controlled Release	1843	10.5	Q1
3	ACS Nano	1586	15.8	Q1
4	International Journal of Nanomedicine	1355	6.6	Q1
5	International Journal of Pharmaceutics	1128	5.3	Q1
6	ACS Applied Materials & Interfaces	1065	8.3	Q1
7	Advance Drug Delivery Reviews	951	15.2	Q1
8	Advance Materials	886	27.4	Q1
9	Hepatology	869	12.9	Q1
10	Cancer Research	822	12.5	Q1

The dual-map overlay of journals visualizes the citation relationships between citing and cited journals, as well as the knowledge flow between disciplines at the journal level by overlaying citing and cited journals in a single map [42]. In the Fig. 7C, four main colored paths were identified. The magenta path shows that research published in physics, materials, and chemistry journals is primarily cited by chemistry/materials/physics and molecular/biology/genetics journals. The orange path shows that research published in molecular/biology/immunology journals is mainly cited by molecular/biology/genetics and chemistry/materials/physics journals. This demonstrates frequent knowledge flow and close citation relationships among journals in the field of liver cancer and nanomaterials.

### Analysis of contributions by co-cited references

When two documents appear together in the reference list of a third citing document, these two references form a co-citation relationship. In the field of liver cancer and nanomaterials research, there are a total of 58,438 co-cited references. Table 7 shows the top 10 most co-cited references. Among them, three studies are from the Icahn School of Medicine at Mount Sinai in the United States, two are from the University of Barcelona in Spain, and others are from the American Cancer Society, the Institute of Chemical Technology in India, National Taiwan University Hospital in China, Tel Aviv University in Israel, and Mayo Clinic in the United States. The most co-cited article is "Global Cancer Statistics" by Jemal A et al., published in 2011 in CA: A Cancer Journal for Clinicians (2023 IF = 503.1, Q1), with 131 co-citations. Among the top five co-cited articles, two are authored by Forner A. from the University of Barcelona, published in 2012 and 2018 in The Lancet (2023 IF = 98.4, Q1). Notably, 80% of the top 10 most co-cited references are review articles. Eight of these are directly related to liver cancer, laying the theoretical foundation for liver cancer research, while the other two discuss the diagnosis and treatment of HCC using nano-carriers. These articles are all published in high-quality journals (Q1). Additionally, Table 8 shows the top 7 centralities of cited references in the field of nanomaterials for liver cancer. The reference with a centrality of 0.1, titled "Adjuvant sorafenib for hepatocellular carcinoma after resection or ablation (STORM): a phase 3, randomised, double-blind, placebo-controlled trial," published in The Lancet Oncology, plays a significant bridging role in the field. It may have accelerated the clinical application of drug delivery using nanomaterials in liver cancer treatment.

**Table 7:** The top 10 co-cited references on nanomaterials for liver cancer research

Rank	Title	Year	Country	Institution	First author	Citation	Journal IF (2023)	JCR partition
1	Global cancer statistics	2011	USA	American Cancer Society	Ahmedin Jemal	131	CA: A Cancer Journal for Clinicians (503.1)	Q1
2	Sorafenib in Advanced Hepatocellular Carcinoma	2008	USA	Icahn School of Medicine at Mount Sinai	Josep M. Llovet	95	New England Journal of Medicine (96.2)	Q1
3	Hepatocellular Carcinoma	2012	Spain	University of Barcelona	Antoni Forner	65	Lancet (98.4)	Q1
4	Hepatocellular carcinoma	2018	Spain	University of Barcelona	Antoni Forner	58	Lancet (98.4)	Q1
5	Hepatocellular Carcinoma	2019	USA	Icahn School of Medicine at Mount Sinai	Augusto Villanueva	52	New England Journal of Medicine (96.2)	Q1
6	Asialoglycoprotein Receptor Mediated Hepatocyte Targeting—Strategies and Applications	2015	India	Institute of Chemical Technology	Anisha A. D'Souza	52	Journal of Controlled Release (10.5)	Q1
7	Efficacy and Safety of Sorafenib in Patients in the Asia–Pacific Region with Advanced Hepatocellular Carcinoma: A Phase III Randomised, Double-Blind, Placebo-Controlled Trial	2009	China	National Taiwan University Hospital	Ann-Lii Cheng	47	Lancet Oncology (41.6)	Q1
8	Hepatocellular Carcinoma: Diagnosis and Management	2009	USA	Icahn School of Medicine at Mount Sinai	Josep M. Llovet	47	CA: A Cancer Journal for Clinicians (503.1)	Q1
9	Nanocarriers as an Emerging Platform for Cancer Therapy	2007	Israel	Tel Aviv University	Dan Peer	45	Nature Nanotechnology (38.1)	Q1
10	A global view of hepatocellular carcinoma: trends, risk, prevention and management	2019	USA	Mayo Clinic	Ju Dong Yang	44	Nature Reviews Gastroenterology & Hepatology (45.9)	Q1

**Table 8:** The top 7 centralities of co-cited references on nanomaterials for liver cancer research

Rank	Title	Year	Country	Institution	First Author	Centrality	Journal IF (2023)	JCR partition
1	Adjuvant sorafenib for hepatocellular carcinoma after resection or ablation (STORM): A phase 3, randomised, double-blind, placebo-controlled trial	2015	Spain	University of Barcelona	Jordi Bruix	0.1	Lancet Oncology (41.6)	Q1
2	The EPR effect: Unique features of tumor blood vessels for drug delivery, factors involved, and limitations and augmentation of the effect	2011	Japan	Sojo University	Jun Fang	0.09	Advanced Drug Delivery Reviews (15.2)	Q1
3	Pazopanib with 5-FU and oxaliplatin as first-line therapy in advanced gastric cancer	2017	China	University of Hong Kong	N.K. Mohamed	0.07	International Journal of Cancer (5.7)	Q1
4	LncRNA PTAR Promotes EMT and Invasion-Metastasis in HCC by Competitively Binding miR-101-3p to Regulate ZEB1 Expression	2018	China	The First Affiliated Hospital of Xi’an Jiaotong University	Wudong Duan	0.07	Drug Design, Development and Therapy (4.7)	Q1
5	Cancer incidence and mortality worldwide: Sources, methods and major patterns in GLOBOCAN 2012	2015	United Nations	International Agency for Research on Cancer	Jacques Ferlay	0.06	International Journal of Cancer (5.7)	Q1
6	Combination of riboflavin and UV-A for Photodynamic Therapy against clinical isolates of Staphylococcus aureus in planktonic and biofilm phases	2014	South Korea	Kyungpook National University	J.E. Chang	0.06	Journal of Photochemistry and Photobiology B: Biology (3.9)	Q1
7	Nanoscale metal–organic frameworks for combined photodynamic and photothermal therapy under near-infrared irradiation	2017	China	Tsinghua University	Ruibo Zhao	0.05	Biomaterials (12.8)	Q1

**Fig. 8:**
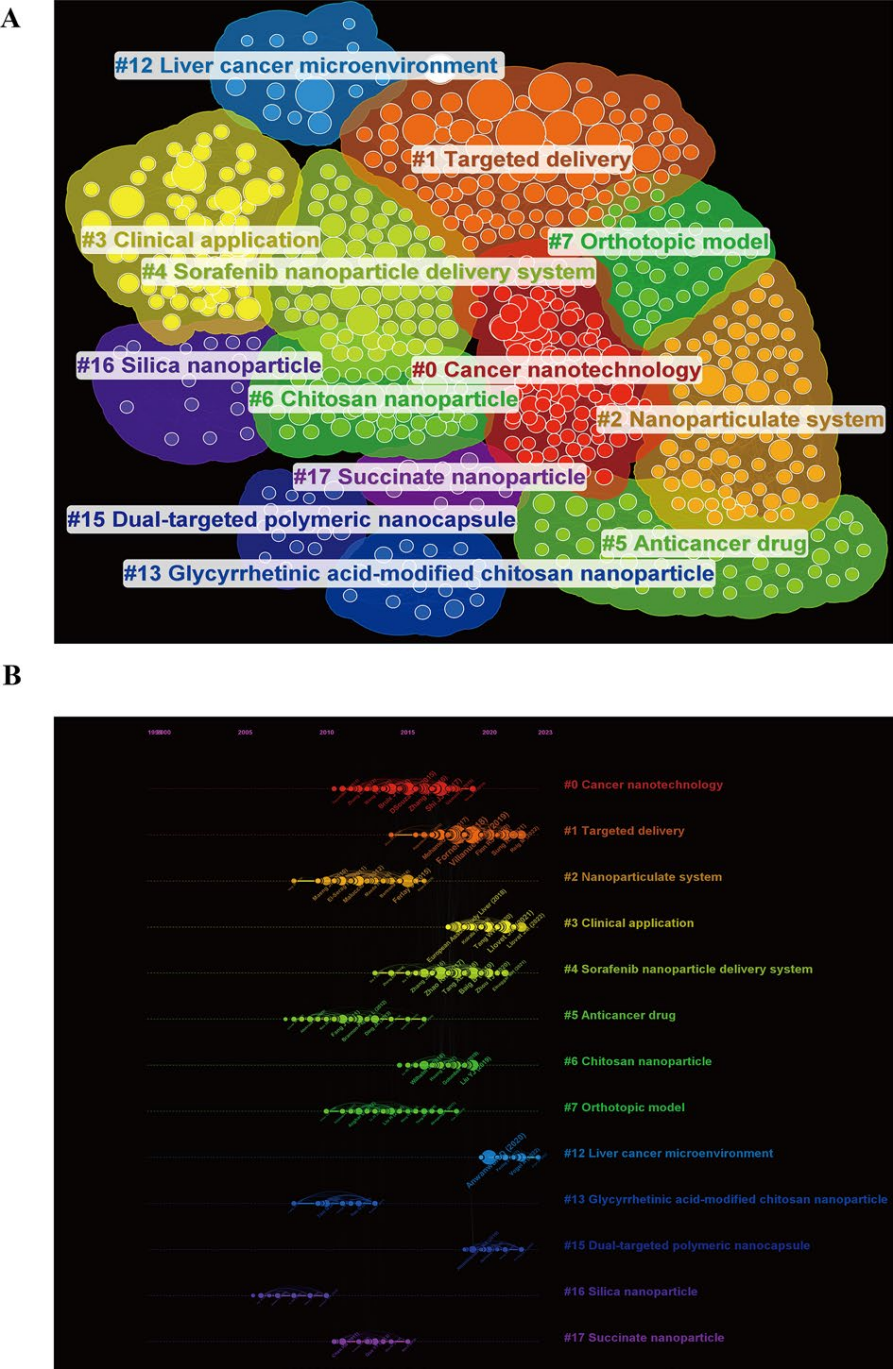
The visualization of co-cited references involved in liver cancer and nanomaterials research. **A** Clustering map of co-cited references. Nodes in the map represent references. The size of the nodes indicates the citation frequency of the references, and their colors and positions reflect the clustering of these references. Different colors represent different clusters, each corresponding to a specific research theme. **B** Timeline view of clusters by co-cited references. The horizontal position of a node indicates the date when the reference first appeared. The size of the node reflects the frequency of its co-citations. Connections between nodes denote co-citation relationships

Cluster analysis is a method of categorizing literature that can reveal major research themes and directions in a large body of references. Using CiteSpace, we categorized these co-cited references into 13 different colored clusters based on title words (Q = 0.7845, S = 0.8761). As shown in the Fig. 8A, each cluster represents a specific research theme. The smaller the number, the more publications the cluster contains. For example, the largest cluster, #0, is named "Cancer Nanotechnology," indicating that the references within this cluster are heavily cited by research on cancer nanotechnology. However, many highly co-cited articles primarily come from cluster #1, "Targeted Delivery" (Fig. 8A).

The timeline view, combining clustering and time-slicing techniques, intuitively displays the evolution trends and interrelationships of research themes over time, helping to identify important research hotspots and key influential papers. In the Fig. 8B, we have drawn a timeline view of co-cited references based on title words. The analysis found that "#16 Silica nanoparticle" is a relatively early research hotspot. Since 2015, the application of nanomaterials in liver cancer has gradually developed towards drug delivery, with increasing attention to "#1 Targeted delivery," "#4 Sorafenib nanoparticle delivery," and "#6 Chitosan nanoparticle." After 2020, the research focus has shifted more towards "#3 Clinical application," with attention to "#12 Liver cancer microenvironment" during the application process, and the design of an advanced drug delivery system, "#15 Dual-targeted polymeric nanocapsule."

When a reference experiences a sudden surge in citations over a specific period, it is known as a citation burst. This phenomenon usually reflects the importance or novelty of the reference. Using CiteSpace, we identified 10 references exhibiting citation bursts in chronological order, with a minimum burst duration of 2 years (Table 9). Duration indicates the start and end years of the citation burst, while strength represents the intensity of the citation change. The red line shows the duration of the citation burst. The dark blue line represents the total citation span from 2004 to 2023, and the light blue indicates periods when the reference did not appear or was not cited. The citation strengths of these 10 references range from 7.65 to 10.9, with durations of 2–5 years. Among them, the references titled "Global Cancer Statistics 2020: GLOBOCAN Estimates of Incidence and Mortality Worldwide for 36 Cancers in 185 Countries" and "Hepatocellular carcinoma" by Llovet JM et al. showed the strongest citation bursts (Strength = 10.9) from 2022 to 2023, indicating that these two references have laid a significant theoretical foundation in the fields of liver cancer and nanomaterials in recent years.

**Table 9:**
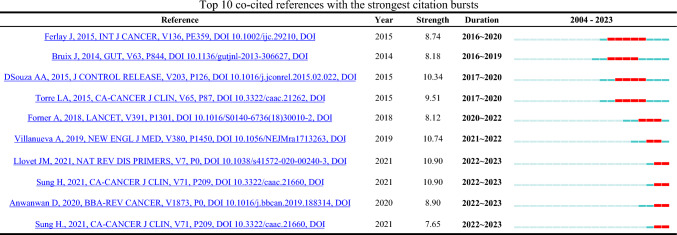
Top 10 co-cited references with the strongest citation bursts

### Analysis of keyword trends and interconnections

Keyword co-occurrence analysis is an important method in bibliometrics for identifying research hotspots and popular topics. Given that the research topics of this study are "liver cancer" and "nanomaterials," these specific terms were excluded when creating the keyword visualization map. Table 10 shows the 20 most frequently occurring keywords in this study, with "Nanoparticles" appearing most frequently (165), indicating its significance as a research direction in the field of nanomaterials for liver cancer. Following this are "Apoptosis" (95), "Drug Delivery" (86), "Sorafenib" (75), and "Doxorubicin" (70).

**Table 10:** The top 20 most cited references on nanomaterials for liver cancer research

Rank	Keyword	Co-occurrence
1	Nanoparticles	165
2	Apoptosis	95
3	Drug delivery	86
4	Sorafenib	75
5	Doxorubicin	70
6	Cytotoxicity	52
7	Chemotherapy	44
8	Nanomedicine	43
9	Photothermal therapy	40
10	Gold nanoparticles	38
11	Curcumin	32
12	Immunotherapy	28
13	Magnetic resonance imaging	28
14	Photodynamic therapy	28
15	Hepg2 cells	27
16	Oxidative stress	27
17	Combination therapy	26
18	Paclitaxel	26
19	Targeted delivery	26
20	Hepg2	25

**Fig. 9:**
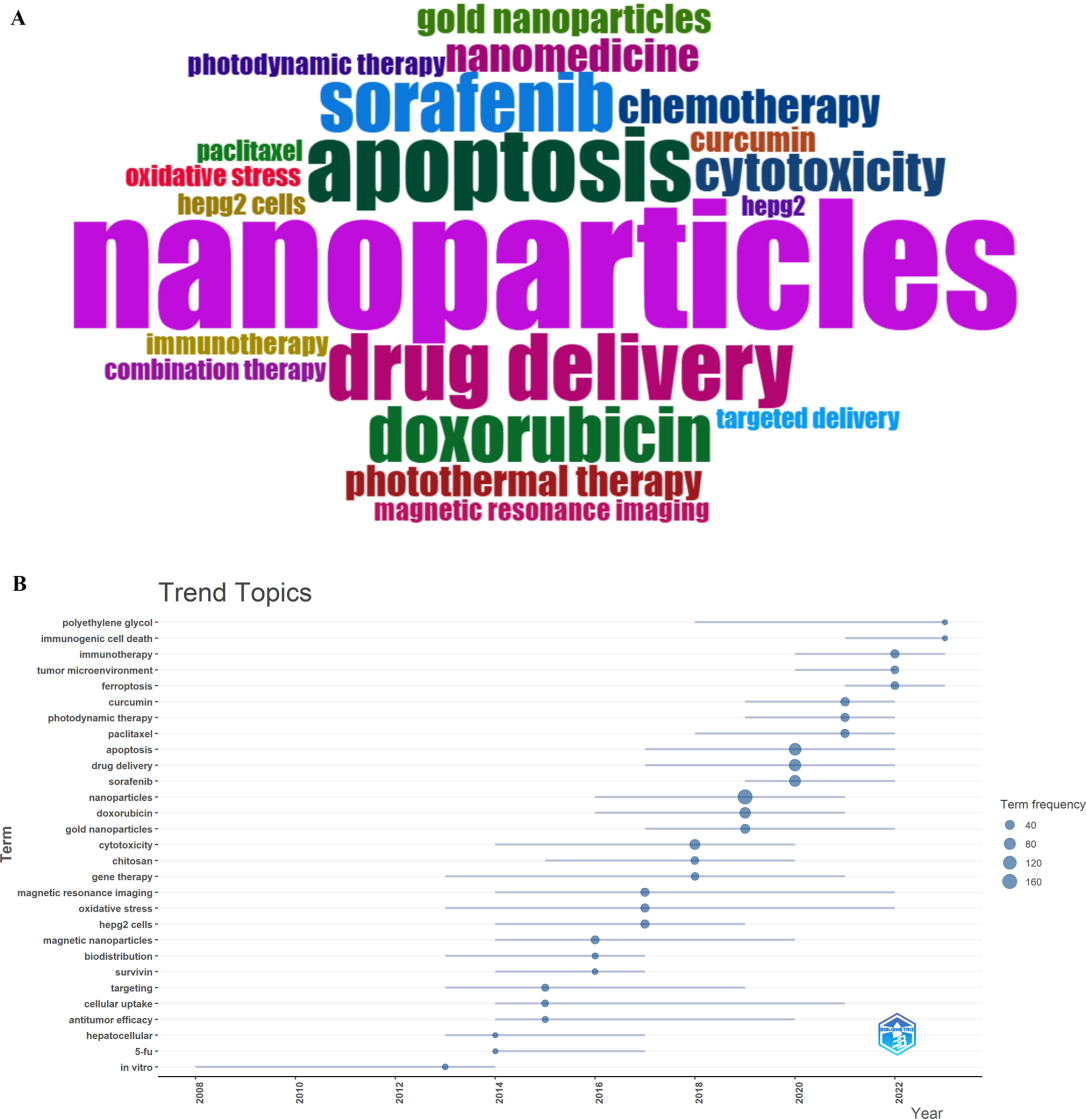
The visualization of keywords involved in liver cancer and nanomaterials research. **A** Word cloud of frequently used keywords. **B** Annual trend chart of keywords changes. The frequency of each keyword is represented by dots of varying sizes. The larger the dot, the higher the frequency of the keyword. The horizontal axis represents the years, while the vertical axis lists the keywords

We created a word cloud based on these top 20 keywords (Fig. 9A) and further filtered keywords with a minimum frequency of 5 occurrences and an average annual frequency of 3 occurrences for a trend evolution analysis (Fig. 9B). As shown in the Fig. 8B, early research (before 2014) mainly focused on in vitro studies, exploring the basic experimental aspects of nanomaterials in biology. Starting in 2014, research gradually delved into biological interactions, with keywords like "cellular uptake," "oxidative stress," and "cytotoxicity" becoming prominent, indicating an increased focus on therapeutic applications and the mechanisms of nanomaterials. Recent studies have shown a surge in interest towards more advanced topics such as "drug delivery," "tumor microenvironment," "photodynamic therapy," "Polyethylene Glycol," and "Immunogenic Cell Death," suggesting that the field is evolving towards more complex and targeted therapeutic approaches.

Additionally, we identified the top 20 keywords with the strongest citation bursts from 2004 to 2023 (Table 11). Among them, "in vivo" had the highest burst strength (14.02) between 2012 and 2017, indicating a peak period for in vivo studies. In recent years, researchers have gradually shifted their focus to keywords such as "Radiofrequency ablation tumor," "Microenvironment," and "Sorafenib." These keywords are still experiencing citation bursts, indicating that they remain hot topics in liver cancer treatment.

**Table 11:**
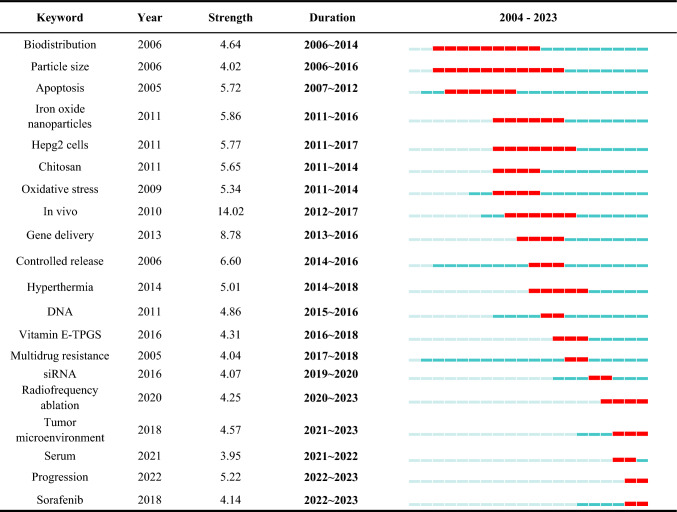
Top 20 keywords with the strongest citation bursts

Keyword analysis not only helps us identify the main research hotspots and themes in the field of nanomaterials for liver cancer but also reveals the evolution trends of research directions and potential frontier areas. This provides important references for future research, enabling researchers to better grasp research trends and optimize their research strategies.

## Discussion

Since the twenty-first century, the application of nanomaterials in the treatment of tumors, especially liver cancer, has significantly increased. With continuous technological innovation and the gradual expansion of application scope, nanomaterials have brought revolutionary changes to the diagnosis and treatment of liver cancer. This study is the first to conduct a comprehensive bibliometric analysis of publications related to the application of nanomaterials in the diagnosis and treatment of liver cancer from January 1, 2004, to December 31, 2023. We conducted a search in the WoSCC and manually screened 1641 relevant publications. To ensure the accuracy of the analysis, we detailed the inclusion and exclusion criteria and excluded duplicate documents. To the best of our knowledge, this is the first comprehensive bibliometric analysis of publications in the field of nanomaterials for the diagnosis and treatment of liver cancer over the past two decades.

From the annual trend of publications, research from 2004 to 2010 mainly focused on the preliminary development of nanomaterials for liver cancer detection and treatment. These studies explored novel fluorescent-labelled NPs, silver NPs, and carbon nanotubes, while also investigating surface modifications to enhance the targeting and selectivity of nanomaterials for liver cancer [43–46]. From 2011 to 2017, the research focus shifted to the application of nanomaterials in liver cancer imaging and therapy. For example, researchers developed biosynthetic NPs and smart-responsive gold NPs and studied their effects on cell signaling pathways and gene expression [47, 48]. Additionally, this period saw the development of biocompatible NPs and magnetic-plasmonic nanocomposites to enhance liver cancer treatment in vivo [49, 50]. Starting in 2018, as researchers recognized the immense potential of nanomaterials in liver cancer diagnosis and treatment, the number of related publications increased rapidly, attracting more academic attention. By the end of 2023, research focused on developing multifunctional nanomaterials for liver cancer detection, imaging, and therapy, such as pH-responsive iron oxide nanoclusters and ultrasound-activated Janus Au-MnO NPs [51, 52]. Simultaneously, this period saw the exploration of functionalized siRNA NPs to significantly improve delivery efficiency and targeted therapeutic effects [53]. Additionally, research extensively explored the combination of nanomaterials with other therapeutic methods. The application of nanomaterials in modulating the TME also emerged as a potential research hotspot [54, 55]. It is noteworthy that the number of publications in 2022 and 2023 was nearly the same, indicating saturation. This could be due to the necessity for extensive experiments and clinical validation to confirm the efficacy and safety of nanomaterials in medical applications. If technical challenges in the synthesis, functionalization, targeting, or toxicity of nanomaterials are difficult to overcome, research progress may be hindered, resulting in fewer publications. Currently, substantial data and findings have been accumulated in the field of nanomaterials for liver cancer diagnosis and treatment. The research focus may be shifting from basic research to clinical application and commercialization, which could lead to a decrease in academic publications but does not necessarily indicate a reduction in research activity.

In terms of country and region distribution, China has made the highest academic contribution to liver cancer and nanomaterials research over the past two decades. This is reflected in the number of publications, centrality, and H-index. Although China's citation per publication is relatively low, it has the highest total citations. The reasons for this include China's large population and high incidence of liver cancer [56]. China's strong economic power and significant investment in healthcare have also promoted the development of this field. National economic support and international cooperation will continue to drive comprehensive progress in this area. In addition to China, developed countries also dominate this field. For instance, although the United States and South Korea have fewer publications (185 and 50, respectively), their citation per publication is quite high (38.91 and 47.82, respectively).

As academic achievements in liver cancer and nanomaterials research continue to increase globally each year, international collaboration has become a pivotal force in advancing this field. These collaborations have not only facilitated the joint development of advanced nanomedicine systems but also strengthened technological integration across nations, particularly in nanoparticle synthesis, materials science, and preclinical animal testing. Regular cross-cultural exchanges and shared resource platforms have further accelerated innovation, streamlining the translation of research findings into potential clinical applications. For example, the collaboration between Poland and China has made notable progress in the development of novel nanoplatforms, combining chemotherapy with PTT to improve liver cancer treatment [57]. Similarly, the cooperation between Iraq and China has utilized platinum NPs to enhance tumor radiosensitivity and improve chemotherapy efficacy [58]. Furthermore, the tripartite collaboration between the United States, China, and Egypt has developed a dual-targeted nanodrug delivery system, which has significantly increased the precision and effectiveness of liver cancer treatment [59, 60]. These examples underscore the global relevance of nanomaterials in liver cancer therapy. Through international cooperation, research teams from diverse countries have contributed uniquely to materials science, drug delivery, and the application of natural compounds. The research outcomes resulting from these collaborations have not only significantly improved therapeutic efficacy and reduced side effects on healthy tissues but also demonstrate broad applicability, benefiting not only high-income countries but also extending to resource-limited regions.

Given that China leads in the number of publications worldwide, the top 10 institutions and authors in the field of liver cancer and nanomaterials research are almost entirely from China. Notably, the work of the Chinese Academy of Sciences and its former researcher Shao D has shown outstanding influence in the field, with the highest total citations, citation per publication, and H-index. In terms of journals, the International Journal of Nanomedicine, ACS Applied Materials & Interfaces, and Biomaterials are the main platforms for publishing research in this field. The International Journal of Nanomedicine not only has the highest publication count but also boasts the highest H-index. Meanwhile, Biomaterials ranks top in total citations, citation per publication, and the 2023 journal impact factor. Additionally, the most frequently co-cited journals are high-impact Q1 journals, further demonstrating their significance and contribution to liver cancer and nanomaterials research.

The co-cited references identified in this study mostly relate to the pathogenesis and pharmacotherapy of liver cancer. They primarily explore the epidemiology, diagnosis, treatment, and molecular characteristics of HCC, indicating their significance in the field of liver cancer. For instance, the most cited reference, "Global cancer statistics," provides an overview of the global cancer burden, noting the rising incidence and mortality rates of liver cancer and proposing measures to mitigate this burden [61]. Studies by Llovet JM et al. and Cheng AL et al. [62, 63] emphasize the efficacy and safety of Sorafenib in advanced HCC and among HCC patients in the Asia–Pacific region. Two review articles by Forner et al. [64, 65] discuss the epidemiology and latest treatment advances of HCC, highlighting the importance of early detection and multidisciplinary treatment. Villanueva A's review article [66] explores the molecular characteristics of HCC and their implications for personalized therapy. Yang et al. [67] summary the global trends, risk factors, prevention, and management strategies of HCC, underscoring the importance of international collaboration. Additionally, these references discuss the applications and advantages of nanomaterials in hepatocyte targeting and drug delivery. D'Souza AA et al. [68] focus on hepatocyte targeting strategies mediated by asialoglycoprotein receptors and their applications in drug delivery. Peer et al.'s review article [69] introduces the rise of nanocarriers as a new platform for cancer therapy, detailing their advantages and challenges in drug delivery. Therefore, these co-cited references indicate the potential of nanomaterials in enhancing drug efficacy and targeted therapy, underscoring their significant research and application value.

Keyword analysis shows that research hotspots of nanomaterials in liver cancer focus on improving drug delivery efficiency, inducing cancer cell apoptosis, PDT and PTT therapy, and combination therapy. As a core technology, NPs are widely used in drug delivery systems by optimizing drug targeting and bioavailability, and reducing side effects. For example, gold and lipid NPs have been studied for targeted drug delivery and gene therapy. Specific drugs, such as doxorubicin (DOX) and sorafenib, combined or loaded with these NPs, significantly enhance efficacy and targeting [48, 70, 71]. NPs can also promote apoptosis by activating apoptotic signaling pathways and increasing oxidative stress [72, 73]. PTT uses the photothermal effect of NPs to kill cancer cells by generating high temperatures [74]. Moreover, the application of NPs in combination therapy has attracted much attention, as combining multiple therapeutic approaches improves overall treatment efficacy and reduces the side effects of single therapies [75]. Studies on the cytotoxicity and effects of NPs on oxidative stress provide important bases for safety evaluation, laying a solid foundation for future clinical applications.

Over the past decade, various emerging therapeutic methods and novel NPs with responsive NDDS have been increasingly applied to the treatment and diagnosis of liver cancer (Table 12). In the overall treatment of liver cancer, early and accurate diagnosis plays a crucial role in improving patient outcomes. Early detection of liver cancer not only significantly increases the chances of a cure but also expands the range of options for surgical or localized therapies, ultimately prolonging survival rates. However, despite the importance of diagnosis in liver cancer management, our bibliometric analysis reveals a clear lack of research focused on diagnostic approaches within the field of nanomaterials for liver cancer. The keyword analysis demonstrates that current studies are predominantly directed towards therapeutic applications, with terms related to drug delivery, chemotherapy, and PTT frequently appearing, while diagnostic-related terms remain relatively scarce. This suggests that the research focus in this field continues to prioritize enhancing therapeutic efficacy through nanomaterials, with comparatively less attention paid to their diagnostic potential.

**Table 12:** Various nanomaterials in liver cancer treatment: mechanisms and applications

Research focus	Type of nanomaterial	Treatment method	Detailed mechanism
Targeted drug delivery	Gold nanoparticles	Targeted drug delivery systems	Gold nanoparticles can be conjugated with antibodies or small molecule drugs to specifically target receptors on liver cancer cells. This enables direct drug delivery to tumor cells, reducing impact on normal cells. Gold nanoparticles can also be engineered to optimize drug release rates through surface modifications
Photothermal therapy	Graphene nanosheets	Near-infrared light-induced tumor heating	Graphene exhibits strong absorption in the near-infrared region, efficiently converting light energy into heat. When graphene nanosheets accumulate in tumor tissues and are exposed to near-infrared light, they generate sufficient heat to cause thermal damage and death of tumor cells
Immunotherapy	Lipid nanoparticles	Delivery of immunomodulators to enhance anti-tumor immune response	Lipid nanoparticles can carry and protect immunomodulators like CpG oligodeoxynucleotides, promoting the maturation and activation of dendritic cells, enhancing the body's recognition and attack on tumor cells. Additionally, these nanoparticles can be engineered with surface modifications to enhance targeting capabilities, ensuring effective concentration of the drug within the tumor microenvironment
Targeted gene therapy	Liposomes	Delivery of CRISPR/Cas9 system for liver cancer-related gene repair	Liposomes use their unique bilayer structure to encapsulate CRISPR/Cas9, protecting these sensitive molecules from degradation by bodily enzymes. With specific ligand modifications, these liposomes can be directed towards liver cancer cells, achieving efficient delivery of gene editing tools and precise repair of tumor genes
Ultrasound imaging and treatment	Microbubbles and nanobubbles	Enhance ultrasound imaging clarity and perform localized drug delivery	Microbubbles and nanobubbles serve as ultrasound contrast agents, producing significant acoustic responses under ultrasound, enhancing imaging contrast of tumor tissues. Additionally, they can be used as drug carriers, where drug release is precisely controlled by ultrasound-guided bubble rupture, enabling precise localized treatment
Targeted drug delivery	Polymeric nanoparticles	Drug delivery and sustained release systems	Polymeric nanoparticles protect drugs from degradation, enhance loading capacity, and stability, achieving sustained and targeted drug delivery. Biodegradable polymers like PLA, PLGA, and PAA are widely used in cancer treatment
MRI and therapy	Magnetic nanoparticles	Used in MRI and drug delivery	Magnetic nanoparticles, due to their superparamagnetic behavior, can serve as contrast agents for MRI and be guided by external magnetic fields to achieve targeted drug delivery and local hyperthermia
Photothermal therapy and drug delivery	Carbon-based Nanomaterials	Used in photothermal therapy, drug delivery, and gene therapy	Including graphene oxide, carbon nanotubes, fullerenes, and carbon dots, these materials have excellent physicochemical properties for photothermal therapy, drug delivery, and gene therapy, with high targeting ability and good biocompatibility
Bioimaging and diagnosis	Quantum dots	Used in early tumor detection and real-time imaging	Quantum dots, like zinc sulfide and indium phosphide systems, are used for early tumor detection and real-time imaging due to their excellent optical properties, improving diagnostic accuracy

Nevertheless, the potential of nanomaterials in early diagnosis should not be underestimated. Nanomaterials can significantly enhance the sensitivity and resolution of imaging techniques, enabling earlier tumor detection. A multicenter, open-label, single-arm phase II clinical trial (NCT03407495) evaluated the effectiveness of a novel superparamagnetic iron oxide nanoparticle (IOP injection) as an MRI contrast agent [20]. A total of 52 patients diagnosed with HCC participated in the study, and all underwent IOP-enhanced MRI scans prior to hepatic resection surgery. The results demonstrated that IOP injection was highly effective in diagnosing HCC, with a sensitivity of 100% calculated per patient and 96% per lesion. IOP-enhanced MRI significantly improved contrast between lesions and surrounding liver tissue, even enabling visualization of small vascular invasions. The safety profile of IOP injection was favorable, with no serious adverse events reported. The study concluded that IOP injection is a promising and safe MRI contrast agent, particularly effective in detecting small or well-differentiated tumors. Moreover, due to the demand for precise localization and complete resection of tumors during HCC surgery, traditional intraoperative imaging techniques face limitations in detecting small lesions and delineating tumor boundaries. A new ultra-stable, homogenous Lipiodol-nanoICG formulation (ChiCTR2200058803), combined with fluorescence laparoscopy, has been developed to improve intraoperative tumor imaging accuracy [76]. The results indicate that this technology significantly enhances intraoperative tumor visualization, showing notable improvements in stability, fluorescence characteristics, and targeting capabilities. It demonstrates exceptional performance in identifying small lesions and tumor margins, addressing the limitations of traditional imaging techniques. Therefore, these nanomaterials used in imaging technologies have been validated in multiple clinical studies and, in certain applications, have received regulatory approval, gradually being integrated into actual diagnostic processes.

In addition to research on the use of nanomaterials for liver cancer diagnosis being primarily focused on improving imaging techniques, recent years have also seen a growing interest in utilizing NPs to target and label specific biomarkers. This emerging trend highlights not only the potential of nanotechnology in enhancing imaging resolution but also its capability to provide more precise molecular diagnostics. Alpha-fetoprotein (AFP) is currently the primary serum biomarker used for the diagnosis of HCC, yet its sensitivity and specificity are somewhat limited. To improve AFP detection, fluorescence-based nanotechnology methods have been widely employed. These methods use fluorescent NPs, such as quantum dots or palladium NPs, to label AFP, combining this with mechanisms like Förster Resonance Energy Transfer (FRET) to achieve efficient molecular detection [77]. Additionally, signal amplification strategies, such as enzyme-free catalytic hairpin assembly (CHA), can further enhance detection sensitivity [78]. CHA is triggered by a target molecule, causing the opening of DNA or RNA hairpin structures, which, in turn, initiates a chain hybridization reaction that amplifies the detection signal without the need for enzymes [79]. When CHA is combined with fluorescent NPs, CHA amplifies the signal of the target molecule, while the fluorescent NPs enhance the fluorescence signal during detection. The synergistic effect of both significantly boosts AFP detection signals while reducing background noise, resulting in a substantial improvement in detection sensitivity and specificity, particularly for early-stage liver cancer screening [78]. Although nanomaterial-based detection technologies for liver cancer biomarkers have shown significant advantages in research, their widespread clinical application still faces numerous challenges. These challenges include a lack of standardized protocols, potential safety and biocompatibility concerns, instability and uneven distribution of nanomaterials within the body, high detection costs, and complex regulatory approvals. Furthermore, the lack of sufficient clinical validation data limits the large-scale clinical adoption of this technology. Based on our bibliometric analysis, the potential of nanomaterials in liver cancer diagnosis—particularly for molecular diagnostics through the detection of specific cancer biomarkers—remains largely unexplored, with many underdeveloped areas of research. Advancing the use of nanomaterials in the early diagnosis of liver cancer could significantly improve early detection outcomes and patient prognoses.

Despite the significant potential of nanomaterials, particularly in enhancing drug delivery and improving therapeutic outcomes, traditional NPs still face numerous technical and biological challenges in practical applications. These challenges include poor stability in vivo, short circulation time, inadequate accumulation in liver cancer tissues, limited penetration, and incomplete drug release [80, 81]. Complicating matters further, the complex microenvironment of liver cancer often limits the efficacy of these therapies [81]. To overcome these challenges, researchers have developed responsive NDDS based on the characteristics of the liver cancer microenvironment, such as pH-responsive, enzyme-responsive, and redox-responsive systems. These systems leverage specific features of the liver cancer microenvironment to achieve precise drug release. pH-responsive systems utilize the acidic nature of the TME to release drugs in low pH conditions. Enzyme-responsive systems leverage the high expression of specific enzymes in tumor cells to release drugs through enzymatic catalysis. Redox-responsive systems use high levels of reducing substances, such as glutathione, in tumor cells to trigger drug release. These systems can achieve precise drug release in liver cancer tissues, cells, or subcellular organelles, increasing the effective concentration of drugs at the target site and significantly enhancing therapeutic efficacy. Moreover, microenvironment-regulated NDDS not only precisely release drugs but also enhance the efficacy of antitumor drugs by modulating the tumor microenvironment.

Currently, although the clinical application of nanomaterials in the treatment of liver cancer remains in its early stages, and the number of related clinical trials is relatively limited, existing research has demonstrated their immense potential and promising efficacy. For instance, a multicenter randomized phase II clinical trial evaluated the efficacy and safety of mitoxantrone-loaded polybutylcyanoacrylate nanoparticles (DHAD-PBCA-NPs) in patients with uHCC [82]. The results showed that, when administered via intravenous infusion, DHAD-PBCA-NPs significantly increased the objective response rate (10.5% vs. 0%) and extended the median overall survival (5.46 months vs. 3.23 months) compared to conventional mitoxantrone injections. Furthermore, the DHAD-PBCA-NPs group demonstrated fewer toxic reactions, particularly with a significantly lower incidence of hematologic adverse events. This suggests that mitoxantrone-loaded nanoparticles present a favorable outlook for the treatment of advanced HCC. Additionally, another study investigated the efficacy of transarterial chemoembolization (TACE) combined with a sorafenib nanoparticle drug delivery system (Ab-SFB-NP) specifically for HCC patients with microvascular invasion [83]. Patients were divided into two groups: one group received TACE combined with the Ab-SFB-NP system, while the other group received TACE combined with a conventional non-nanoparticle delivery system. Three months post-treatment, the disease control rate in the Ab-SFB-NP group was significantly higher than in the control group. Moreover, the incidence of adverse effects in the experimental group was relatively lower, with manageable side effects such as hand-foot syndrome and diarrhea. The study concluded that the combination of TACE and the Ab-SFB-NP system provided superior clinical benefits in disease control, further underscoring the potential of nanoparticle-based delivery systems in liver cancer treatment. Although the number of clinical trials remains limited, existing findings provide strong support for the application of nanotechnology in liver cancer therapy. With further clinical research, this technology is expected to be more widely utilized in liver cancer treatment, potentially improving therapeutic outcomes and patient prognosis.

Based on our bibliometric analysis of the contributions of various authors, Shao and Wang are pioneers in the application of Janus nanomaterials in this field. These materials possess a dual-sided structure, with one side offering magnetic or optical properties and the other designed for drug loading, enabling simultaneous targeted delivery and therapeutic action. In the highly cited article by Shao et al. [84] published in Biomaterials in 2016, titled "Janus 'nano-bullets' for mag netic targeting liver cancer chemotherapy," they synthesized a Janus nanocomposite (M-MSNs-DOX) using the sol–gel method. This nanocomposite combines a Fe3O4 magnetic head with a mesoporous silica body loaded with DOX. The magnetic properties of Fe_3_O_4_ allow the particles to be guided to the tumor site through an external magnetic field, while DOX, an anticancer drug, can be released under specific conditions. Using a Sulforhodamine B assay (a staining technique for assessing cell viability) and magnetic guidance, the antitumor effect and systemic toxicity of M-MSNs-DOX were evaluated in cell lines and an H22 HCC model in mice. Results demonstrated that M-MSNs-DOX could respond to pH changes in an acidic environment (such as the tumor microenvironment), releasing less than 5% of DOX at pH 7.4 within 24 h, but releasing up to 50% at pH 5.5 within the same period. Under magnetic guidance, M-MSNs-DOX exhibited significant tumor inhibition effects on various cancer cells (such as HepG2, A549 and SKOV-3) and in a mouse liver cancer model, while significantly reducing the systemic toxicity of DOX in normal cells (such as HL-7702, HUVEC and H9C2), indicating that this material enhances therapeutic efficacy while reducing side effects. Subsequently, Wang and Shao et al. [48] synthesized Folic Acid-Gold-Mesoporous Silica Janus NPs (FA-GSJNs) using a modified sol–gel method. These Janus NPs feature a dual-sided structure, with one side providing folic acid targeting and the other leveraging the photothermal properties of gold NPs. These novel NPs not only achieved drug release based on pH response, utilizing the acidic nature of the TME to trigger drug release, but also combined the photothermal and radiosensitizing effects of gold NPs. Moreover, they could effectively absorb and attenuate X-rays, serving as contrast agents for CT imaging, thereby enhancing the clarity of tumor imaging.

In addition to the extensive use of gold NPs, silver NPs have also demonstrated significant potential in liver cancer therapy. Another study by Wang et al. [85] showed that Janus-type silver-mesoporous silica nanoparticles (FA-JNPs@ICG) exhibited strong antitumor effects when combined with PTT and chemotherapy. PTT involves the use of NPs to convert light energy—typically from near infrared (NIR) light—into heat, which ablates cancer cells through thermal effects. In this study, mice models were divided into three treatment groups. The first group was treated with FA-JNPs@ICG combined with NIR, where FA-JNPs@ICG has a Janus structure, enabling it to integrate multiple functions. The second group was treated solely with indocyanine green (ICG), a dye commonly used in cancer therapy that can absorb light and convert it into heat for PTT. The third group received FA-MSNs@ICG, a singular mesoporous silica nanoparticle, which offers fewer functions compared to FA-JNPs@ICG. These groupings allowed the researchers to compare the tumor inhibition and apoptosis rates across different treatment modalities. In vivo experiments revealed that the FA-JNPs@ICG + NIR group achieved higher tumor inhibition and apoptosis rates (88.9%) compared to ICG or FA-MSNs@ICG alone. This superior performance is likely due to the Janus structure of FA-JNPs@ICG, which enables the integration of multiple functionalities such as radiosensitization, antibacterial properties, and PTT. In contrast, FA-MSNs@ICG, being a singular nanoparticle, lacks these synergistic effects. However, when considering the use of silver NPs in clinical settings, the potential cytotoxicity resulting from silver ion release to healthy cells must be carefully evaluated. Therefore, gold NPs are often used in PTT, radiotherapy, imaging, and drug delivery due to their good biocompatibility and stability. In contrast, silver NPs are used in combined PTT and chemotherapy to release silver ions through the photothermal effect, achieving high tumor inhibition.

Notably, structural variations in nanomaterials significantly influence their physicochemical properties and subsequent applications. In a 2018 study published in *Biomaterials*, Wang Z et al. [86] investigated the differences between spherical and rod-shaped magnetic mesoporous silica nanoparticles (M-MSNs) for use in magnetically-mediated suicide gene therapy targeting HCC. These NPs were injected into HepG2 xenograft nude mouse models to assess tumor inhibition and systemic toxicity. The results revealed that the rod-shaped M-MSNs had a higher drug loading efficiency (42.5%) and drug content (29.8%) compared to spherical M-MSNs (33.7% and 25.2%). Moreover, the rod-shaped particles demonstrated superior antitumor effects in both in vivo and in vitro experiments, especially at higher doses. This indicates that the rod-shaped NPs possess a greater capacity for drug encapsulation and delivery compared to spherical NPs, leading to more effective tumor inhibition, particularly under high-dose conditions. Additionally, nanomaterials of varying shapes display unique properties that may impact their therapeutic potential. For instance, gold triangular nanomaterials exhibit excellent photothermal conversion efficiency, whereby they convert light energy into heat, rendering them highly advantageous for PTT [87]. This material also leverages the hypoxic nature of the tumor microenvironment, making it suitable for a multifunctional treatment approach combining radiation, chemotherapy, and PTT for liver cancer. As the unique physical and chemical properties of nanomaterials are shape-dependent, it is crucial to consider these structural variations when selecting nanomedicine carriers to optimize therapeutic outcomes and minimize side effects. Therefore, these multifunctional nanoplatforms not only enhance drug targeting and therapeutic efficacy but also broaden the application potential of nanomaterials in both tumor diagnosis and treatment, positioning them as promising candidates for multimodal cancer diagnostic and therapeutic platforms.

PDT is increasingly recognized for its promising role in treating HCC. This therapy works by using light to activate specific molecules, leading to selective tumor cell destruction while minimizing damage to healthy tissues. PDT not only directly kills tumor cells but also helps block tumor blood vessels and boosts the body’s immune response. A key component of PDT is the photosensitizer, a molecule that absorbs light at specific wavelengths to produce reactive oxygen species (ROS). These ROS are responsible for triggering either cell death (apoptosis) or cell destruction (necrosis) in the tumor. However, traditional photosensitizers often suffer from aggregation-caused quenching (ACQ), where their fluorescence and ROS production decrease when they clump together. This reduces their effectiveness for both imaging and therapy. To address this, Gao et al. [88] developed photosensitizers with aggregation-induced emission (AIE) properties. AIE photosensitizers emit strong fluorescence when clustered, making them useful for both imaging and therapy in their aggregated form. These new materials were incorporated into targeted nanodots called T-TPETS, which demonstrated excellent ability to target tumor cells and monitor PDT treatment in real-time. Targeted nanodots like T-TPETS help guide treatment and ensure only cancer cells are affected. Both in vitro and in vivo experiments demonstrated that T-TPETS nanodots exhibited excellent tumor-targeting imaging capabilities, facilitating real-time monitoring during PDT, and generated efficient ROS under light irradiation, inducing time- and concentration-dependent cell death. At high PDT intensity, tumor cell necrosis was directly induced; at low PDT intensity, apoptosis was induced via a mitochondrial-mediated pathway. This combination provides strategic and technical basis for the targeted and image-guided PDT of HCC.

Interestingly, although some of the fluorescence quenching phenomena are caused by intramolecular charge transfer (ICT) due to heavy atom effects and strong electron-withdrawing groups (such as nitro groups and halogens), this principle does not apply to all aggregation-induced emission luminogens (AIEgens). Teng et al. [89] designed and synthesized various cyanostyrene (CST)-based AIEgens by introducing different electron donors and fluorescence quenching groups at both ends of the CST "bridge." It was found that CST-based AIEgens containing Br and NO_2_ groups exhibited abnormal fluorescence enhancement, contradicting the traditional quenching fluorescence rules. Additionally, different PSs show significant differences in the efficiency and types of ROS generation. Xu et al. [90] constructed six AIE-active PSs with the same electron acceptor but different electron donors and "π bridges", synthesizing these PSs using classic Suzuki and Knoevenagel reactions. Their chemical structures were confirmed by 1H and 13C NMR to study the ROS efficiency and types of these PSs in different aggregation environments. Results showed that, at the molecular structure level, a low-energy T1 "π-bridges" helps trigger superoxide anion production, while triphenylamine (TPA) as an electron donor is more conducive to hydroxyl radical generation than dimethylamine. At the aggregation level, bovine serum albumin (BSA) significantly improved superoxide anion generation efficiency. Upon activation by light of specific wavelengths, TPABZPy NPs coated with BSA, having TPA donors and benzothiadiazole (BZ) π bridges exhibited excellent PDT effects both in vitro and in vivo. In vitro studies showed that TPABZPy could completely kill cancer cells at a low concentration (2 µM). In vivo studies showed that TPABZPy@BSA NPs effectively inhibited tumor growth with no apparent toxicity in the major organs of mice. Therefore, whether T-TPETS nanodots or TPABZPy@BSA NPs, they represent a strategy of combining PSs with NPs. This combination significantly enhances the stability, targeting, and biocompatibility of PSs, thereby improving the efficacy of PDT. This method has broad application prospects in nanomedicine and cancer therapy.

SDT is an emerging method for treating liver cancer that combines ultrasound with sonosensitizers to generate ROS for effectively killing tumor cells. Similar to PDT, SDT features high selectivity and low side effects, and it can be combined with other treatments such as chemotherapy and radiotherapy to further enhance therapeutic efficacy. Li et al. [91] designed a nanosonosensitizer made of hollow mesoporous organosilicon NPs (HMONs-PpIX-RGD), targeting HCC cells (SMMC-7721) through combined SDT and chemotherapy. Under ultrasound irradiation, HMONs-PpIX-RGD NPs generated ROS, such as singlet oxygen (^1^O2), inducing tumor cell apoptosis. In vitro experiments showed significant anticancer effects even at a very low concentration of DOX (0.5 µg/mL); in vivo mouse models showed a tumor inhibition rate of 84.7% with ultrasound irradiation and no significant toxicity to major organs. Lin et al. [52] used Janus Au-MnO NPs, which dissociate into smaller particles under ultrasound and GSH treatment, directly generating ROS and Mn^2+^. Mn^2+^ further produces more ROS through the Fenton reaction, significantly enhancing tumor inhibition. In vitro experiments showed a 2.2-fold increase in ROS generation and a significant increase in apoptosis rates; in vivo orthotopic liver cancer mouse models showed a significant improvement in tumor inhibition with ultrasound irradiation, a 2.6-fold increase in signal-to-noise ratio, and no significant toxicity to major organs, demonstrating high efficacy and safety in tumor treatment. Although these experimental results show promising therapeutic effects, the findings from mouse models may not fully translate to humans. Additionally, the biodegradation process of NPs in vivo, as well as the potential immune reactions, side effect occurrence rates, and severity need further investigation and confirmation in clinical trials. Therefore, caution is needed when applying these treatments in clinical practice.

## Conclusions

In summary, future research on nanomaterials in the field of liver cancer should gradually focus on the following aspects: (1) conducting larger-scale and longer-term preclinical and clinical studies, including patient selection criteria for different stages, detailed treatment regimen design, and comprehensive analysis of various evaluation indicators; (2) investigating the efficacy and safety of different nanomaterials across diverse patient populations, validating personalized treatment plans through large-scale clinical trials, and thoroughly assessing long-term efficacy and potential side effects; (3) optimizing the preparation and surface modification techniques of nanomaterials to enhance their targeting capabilities and reduce side effects. Developing new nanocarriers and functionalization methods, particularly in the area of targeted drug delivery systems, should be a priority. Further studies are needed to improve the efficiency of nanocarriers in delivering chemotherapy drugs, reduce systemic toxicity, thereby enhancing therapeutic outcomes and minimizing damage to healthy tissues; (4) developing novel nanotherapeutic approaches and combination treatment strategies, and exploring the synergistic effects of combining nanotechnology with immunotherapy or radiotherapy. A deeper understanding of the mechanisms by which NPs enhance the effectiveness of existing therapies will assist in the design of more comprehensive treatment protocols. Multimodal synergy, through combination treatment strategies, has the potential to significantly enhance therapeutic outcomes, and further exploration of specific mechanisms and case studies is warranted; (5) investigating the application of nanomaterials in early cancer diagnosis, particularly in the identification and quantification of cancer biomarkers. Early detection is crucial for successful treatment, and NPs hold the potential to provide more sensitive and specific diagnostic tools, which is particularly important for early screening of liver cancer and other malignancies; (6) exploring the role of nanotechnology in personalized medicine by tailoring treatment strategies to the genetic and molecular characteristics of individual patients’ tumors. Personalized treatment plans can target the unique features of a patient’s cancer, thereby improving therapeutic efficacy. This approach holds significant promise, especially in the diverse treatment strategies required for liver cancer; (7) conducting long-term studies on the biocompatibility and potential toxicity of nanomaterials to ensure their long-term safety in cancer treatment. Such studies are critical not only for patient safety but also for regulatory approval and the clinical translation of nanotechnology; (8) prioritizing the establishment of standardized regulatory frameworks for the clinical application of nanotechnology. These frameworks will not only ensure the safety and efficacy of therapies but also expedite the translation of innovative treatments from laboratory research to clinical practice; (9) fostering interdisciplinary collaboration between nanotechnologists, oncologists, and materials scientists. Such collaboration can leverage the expertise and resources of various fields to address the complex challenges in cancer treatment and promote the development of more comprehensive and innovative therapeutic solutions.

In addition to these research priorities, the findings also carry significant policy implications. Policymakers and funding agencies should fully recognize the growing importance of nanomaterials in cancer treatment, particularly in the context of liver cancer. By supporting research in this area, policy decisions can effectively drive scientific innovation, accelerate the development of novel therapies, and provide a solid foundation for clinical applications. Increased funding for nanotechnology research will not only lead to scientific breakthroughs but will also have profound impacts on healthcare systems, economic outcomes, and patient prognoses, particularly in the areas of personalized medicine and early diagnosis. Continued investment in this field is expected to promote more precise cancer treatments, ultimately improving overall patient health outcomes. Moreover, this study aims to engage a broad range of stakeholders, including researchers, clinicians, and industry leaders, to foster collaboration and drive forward the potential applications of nanomaterials in oncology. By highlighting both diagnostic and therapeutic advances, the findings offer a foundation for further discussions and development in this promising area.

Our study highlights the transformative potential of nanomaterials in the diagnosis and treatment of liver cancer and underscores their critical role in the future of cancer therapy. Through bibliometric analysis, we not only demonstrate the growing research trends and academic interest in this interdisciplinary field but also emphasize the importance of nanomaterials in overcoming the limitations of conventional liver cancer treatments. Additionally, by conducting a comprehensive analysis of the current trends and challenges related to nanomaterials in the field of liver cancer, this study provides a solid theoretical foundation and research direction for students and early-career researchers. Furthermore, by identifying gaps and underexplored areas in existing research, this study encourages the development of more precise, patient-centered treatment strategies, thereby driving the field toward personalized medicine. These findings not only have significant implications for improving clinical outcomes but also promote the advancement of innovative therapeutic approaches, while offering new opportunities for future interdisciplinary collaboration and policy support.

Our study inevitably has some limitations. First, the data were obtained solely from the WoSCC database, which may result in an incomplete literature search and the omission of relevant studies from other databases. Second, we selected only English publications, potentially underestimating the academic contribution of non-English papers. Additionally, manually removing irrelevant papers might have introduced selection bias, while recently published high-quality literature might have been underestimated in the analysis due to fewer citations. Although the WoSCC database is extensive and comprehensive, reflecting the current research status, its coverage still has limitations, and the citation impact is often subject to delays. Therefore, future updates to bibliometric data are necessary to further clarify the scientific trends and research hotspots of nanomaterials in the field of liver cancer.

## Data Availability

Data is provided within the manuscript file.

## Abbreviations

HCC: Hepatocellular carcinoma
ICI: Immune checkpoint inhibitor
TME: Tumor microenvironment
uHCC: Unresectable hepatocellular carcinoma
CAR-T: Chimeric antigen receptor T-cell
PTT: Photothermal therapy
PDT: Photodynamic therapy
SDT: Sonodynamic Therapy
NPs: Nanoparticles
MRI: Magnetic resonance imaging
CT: Computed tomography
SPIONs: Superparamagnetic iron oxide nanoparticles
NDDS: Nanotechnology-based drug delivery systems
EPR: Enhanced permeability and retention
WoSCC: Web of Science Core Collection
JCR: Journal Citation Reports
IF: Impact factor
TLS: Total Link Strength
LS: Link Strength
DOX: Doxorubicin
AFP: Alpha-fetoprotein
FRET: Förster Resonance Energy Transfer
CHA: Catalytic hairpin assembly
DHAD-PBCA-NPs: Mitoxantrone-loaded polybutylcyanoacrylate nanoparticles
TACE: Transarterial chemoembolization
FA-GSJNs: Folic Acid-Gold-Mesoporous Silica Janus Nanoparticles
NIR: Near-Infrared
ICG: Indocyanine Green
PS: Photosensitizer
ROS: Reactive oxygen species
AIE: Aggregation-induced emission
ICT: Intramolecular charge transfer
AIEgens: Aggregation-induced emission luminogens
CST: Cyanostyrene
TPA: Triphenylamine
BSA: Bovine serum albumin
BZ: Benzothiadiazole
